# Identifying network biomarkers of cancer by sample-specific differential network

**DOI:** 10.1186/s12859-022-04772-1

**Published:** 2022-06-15

**Authors:** Yu Zhang, Xiao Chang, Jie Xia, Yanhong Huang, Shaoyan Sun, Luonan Chen, Xiaoping Liu

**Affiliations:** 1 Key Laboratory of Systems Biology, Hangzhou Institute for Advanced Study, University of Chinese Academy of Sciences, Hangzhou, 310024 China; 2 Key Laboratory of Systems Health Science of Zhejiang Province, Hangzhou, 310024 China; 3 School of Mathematics and Statistics, Shandong University, Weihai, 264209 Shandong China; 4 Institute of Statistics and Applied Mathematics, Anhui University of Finance & Economics, Bengbu, 233030 China; 5 Center for Excellence in Molecular Cell Science, Shanghai Institute of Biochemistry and Cell Biology, Chinese Academy of Science, Shanghai, 200031 China; 6 School of Mathematics and Statistics, Ludong University, Yantai, 264025 China; 7 West China Biomedical Big Data Center, Med-X center for informatics, West China Hospital, Sichuan University, Chengdu, 610041 China

**Keywords:** Single-sample-differential-network, Gene-expression-data, Cancer driver-gene, Enrichment analysis

## Abstract

Abundant datasets generated from various big science projects on diseases have presented great challenges and opportunities, which contributed to unfolding the complexity of diseases. The discovery of disease-associated molecular networks for each individual plays an important role in personalized therapy and precision treatment of cancer-based on the reference networks. However, there are no effective ways to distinguish the consistency of different reference networks. In this study, we developed a statistical method, i.e. a sample-specific differential network (SSDN), to construct and analyze such networks based on gene expression of a single sample against a reference dataset. We proved that the SSDN is structurally consistent even with different reference datasets if the reference dataset can follow certain conditions. The SSDN also can be used to identify patient-specific disease modules or network biomarkers as well as predict the potential driver genes of a tumor sample.

**Supplementary Information:**

The online version contains supplementary material available at 10.1186/s12859-022-04772-1.

## Introduction

With the rapid advance of deep sequencing technology for cancer genomes, several large-scale projects, i.e. The Cancer Genome Atlas (TCGA) [1, 2] and International Cancer Genome Consortium (ICGC) [3, 4], were performed to provide opportunities for the comprehensive understanding of molecular mechanisms and pathogenesis underlying cancer [5, 6]. One crucial challenge for cancer omics data sets is to get insight into the mechanism of tumor progression [7–9]. The studies have shown that the molecular mechanisms of most complex diseases were due to the dysfunction of relevant systems/networks instead of the malfunction of single molecules [10–12]. Therefore, constructing a network to analyze molecular mechanisms has become an effective method for studying complex diseases. The dynamic interactions and regulations between molecules [13–19] can detect the causal disease genes/module biomarkers at a single sample level. The edge biomarker’s method [20, 21] calculates the difference between normal network and disease network and discovers a set of differentially correlated gene pairs. Besides, network biomarkers [22] or subnetwork markers [14, 23] can accurately characterize disease states. In actuality, these methods construct the individual-specific network based on the reference network from a group of reference samples. However, it is unclear how can a different reference sample set or the reference network affect the structure of single-sample network, or if or not a different reference sample set can result in a different network structure. In other words, these methods cannot distinguish the consistency of single-sample network with different reference sample sets.

The SSN method [24] estimates the perturbations of Pearson’s correlation coefficient (PCC) for each pair of genes in a single sample, and it can be used to construct the individual-specific network for disease samples and control samples, which called the Disease network and Control network. Compared with the previously described SSN, we establish the SSDN by comparing and finding the difference between the Disease network and Control network. A reference sample set is required to construct a reference network in SSDN, and the consistency of single-sample-Pearson correlation coefficients *(s-PCC)* needs to be considered in the SSDN. For this consideration, we analyzed the conditions of consistency of *s-PCC* based on different reference networks in this work, and proved that the *s-PCC* based on different reference networks are consistent in the following two cases: the number of reference samples is sufficiently large; the reference sample sets follow the same distribution. In other words, provided that if one of these two conditions is satisfied for the reference samples, we have the same SSDN structure, which is independent of the choice of the reference samples. This result provides a theoretical foundation for determining the reference network in the construction of SSDN.

In this work, we first gave the theoretical result on the conditions of the reference samples to construct a consistent SSDN, and then validated the consistency of *s-PCC* based on different reference networks both by simulated data and by three gastric cancer datasets from GEO datasets from the TCGA database. For clarifying the sample-specific characteristics of SSDN, we established a disease-specific sample network (DSSN), which is similar to SSDN but is based on non-paired sample data, to identify potential sample-specific driver genes to assess clinic prognosis information, which is strongly correlated with individual somatic mutation genes and validated by the enrichment analysis. The results of survival analysis for the potential sample-specific driver genes demonstrate that the networks with those genes can be used as effective module biomarkers to predict the prognosis for patients.

## Material and methods

### Data processing

The gene expression profiles for gastric cancer were from the GEO database (http://www.ncbi.nlm.nih.gov/geo/) including datasets GSE27342, GSE63089, and GSE33335. The three datasets contain 80 pairs, 45 pairs, and 25 pairs of RNA-Seq data from gastric cancer tissues and matched adjacent tumor-adjacent tissues from 150 cancer patients. The IDs of probe sets were mapped to the gene symbols. Probe sets without corresponding gene symbols were not considered in this study. All profiles were normalized by the RMA (robust multi-array averaging) methods, and the probe sets were mapped to their corresponding gene symbols. The expression values of replicated probe sets were averaged to one gene. As a result, 17,325 genes were gotten for the following study. In addition, four tumor datasets, which were Breast invasive carcinoma (BRCA), Lung adenocarcinoma (LUAD), Lung squamous cell carcinoma (LUSC), and Liver Hepatocellular Carcinoma (LIHC), were gotten from the TCGA data portal (http://cancergenome.nih.gov). There were 1102 tumor and 113 tumor-adjacent samples in BRCA, 533 tumor and 59 tumor-adjacent samples in LUAD, 502 tumor and 49 tumor-adjacent samples in LUSC, 371 tumor and 50 tumor-adjacent samples in LIHC, and the clinic information of these samples were also downloaded from TCGA. Then, 24,991 mRNAs/genes were obtained for each sample in TCGA and the data of tumor-adjacent tissue was considered as the normal samples for further study. Finally, we obtained 50 tumor samples for BRCA from the ICGC database (International Cancer Genome Consortium, https://icgc.org/) as a follow-up verification.

### Functional enrichment for the individual specific network

The existing cancer genes were gathered from the Cancer Gene Census database [25] (CGC, https://cancer.sanger.ac.uk/census/) and a hypergeometric test was used to calculate the functional enrichment of genes in the SSDN. The formula of the hypergeometric test is:

$$P\left( {X \ge k} \right) = 1 - \sum\limits_{i = 0}^{k - 1} {\frac{{\left( \begin{gathered} K \hfill \\ i \hfill \\ \end{gathered} \right)\left( \begin{gathered} N - K \hfill \\ n - i \hfill \\ \end{gathered} \right)}}{{\left( \begin{gathered} N \hfill \\ n \hfill \\ \end{gathered} \right)}}} ,$$

where *N* is the number of genes of the gene expression profiles, *K* is the number of genes existing cancer-related genes in the CGC database, *n* is the number of genes in the SSDN of a single sample and *i* is the number of overlapped genes between *K* and *n*. *P* is the statistical significance of the hypergeometric test. If *P* < 0.05, then we regarded that the enrichment for the CGC database considered statistically significant. In addition, the enrichment analysis of genes in the SSDN was conducted using DAVID Bioinformatics Tool (version 6.8, https://david.ncifcrf.gov/home.jsp)26 in the cancer pathway from the KEGG (Kyoto Encyclopedia of Genes and Genomes).

### Survival analysis for the individual specific network

To confirm whether the genes from SSDN are related to disease, we used them as a network biomarker to observe the effect between gene expression and survival rate in samples. Here we defined the hub gene, which is a gene that is highly connected with others, or a gene with a high degree. First, we computed the top *m* highest degree genes for SSDN of all samples composing of the hub genes in one cancer. Second, for a single sample, if the top *n* highest degree genes of this sample included half of the hub genes, then the gene was chosen into high-risk group, on the contrary, it would be taken into low-risk group. Survival analysis was performed on the disease samples based on the hub genes. Furthermore, the log-rank test (with *p* < 0.05 considered significantly) in R/Bioconductor [27] was used to evaluate the statistically significant the survival curves between the high and low-risk groups. An independent data from the ICGC database were used to validate our results.

### The theoretical foundation of SSN based on different reference networks

Assume that $X = \left[ {x_{1} , \ldots ,x_{n} } \right]$ and $Y = \left[ {y_{1} , \ldots ,y_{n} } \right]$ are two expression vectors for gene *X* and *Y* in reference samples (reference*1*) with length *n*, where $x_{i}$ is the expression of gene *X* for the *i*th $\left( {1 \le i \le n} \right)$ sample in reference samples*,* and $y_{j}$ is the expression of gene *Y* for the *j*th $\left( {1 \le j \le n} \right)$ sample in reference samples. Here, *n* can be considered as the number of the reference samples, $x_{i}$ and $y_{j}$ represent the expression values of two gene *X* and *Y* in reference samples. And the *PCC* for gene *X* and *Y* can be calculated as follows.

$$R_{n} = \frac{{\sum\limits_{i = 1}^{n} {\left( {x_{i} - \overline{x}_{n} } \right)\left( {y_{i} - \overline{y}_{n} } \right)} }}{{\sqrt {\sum\limits_{i = 1}^{n} {\left( {x_{i} - \overline{x}_{n} } \right)^{2} \sum\limits_{i = 1}^{n} {\left( {y_{i} - \overline{y}_{{\varvec{n}}} } \right)^{2} } } } }}.$$

There were two new samples $S_{a}$ with expression $\left( {x_{a} ,y_{a} } \right)$ and $S_{b}$ with expression $\left( {x_{b} ,y_{b} } \right)$ for gene *X* and *Y*. The two samples were added into reference samples to form new vector pairs $\left[ {\left( {X,x_{a} } \right),\left( {Y,y_{a} } \right)} \right]$ and $\left[ {\left( {X,x_{b} } \right),\left( {Y,y_{b} } \right)} \right]$. Then the *PCC*s between vectors $\left( {X,x_{a} } \right)$ and $\left( {Y,y_{a} } \right)$, between vectors $\left( {X,x_{b} } \right)$ and $\left( {Y,y_{b} } \right)$ with the length (*n* + *1*) were calculated as $R_{na}$ and $R_{nb}$. The differences of *PCC*s between before and after adding the new samples were $\Delta_{na} = R_{na} - R_{n}$ and $\Delta_{nb} = R_{nb} - R_{n}$.

Then we have another two reference vectors $X^{\prime } = \left[ {x_{1}^{\prime } ,x_{2}^{\prime } , \ldots ,x_{m}^{\prime } } \right]$ and $Y^{\prime } = \left[ {y_{1}^{\prime } ,y_{2}^{\prime } , \ldots ,y_{m}^{\prime } } \right]$ with length *m* for another reference samples (reference*2*), where $x_{i}^{^{\prime}}$ is the *i*th $\left( {1 \le i \le m} \right)$ element of gene $X^{\prime }$ and $y_{j}^{\prime }$ is the *j*th $\left( {1 \le j \le m} \right)$ element of gene $Y^{\prime }$ in reference*2.* Here, *m* can be considered to be the number of the reference samples, $x_{i}^{\prime }$ and $y_{j}^{\prime }$ represent the expression levels of two molecules $X^{\prime }$ and $Y^{\prime }$ respectively. When the same two new samples $S_{a}$ and $S_{b}$ added to $X^{\prime }$ and $Y^{\prime }$, the differences of *PCC* are $\Delta_{ma} = R_{ma} - R_{m}$ and $\Delta_{mb} = R_{mb} - R_{m}$.

Derived from our mathematical theory (Note S1), if given the relationship of $\Delta_{na}$ and $\Delta_{nb}$, then the relationship of $\Delta_{ma}$ and $\Delta_{mb}$ got two conclusions: One, assuming $\Delta_{na} > \Delta_{nb}$ based on reference*1*, if $n,m \to \infty$, we can get $\Delta_{ma} > \Delta_{mb}$ based on reference*2,* vice versa. Another, if vector $X$ and vector $X^{\prime}$ belong to one independent identically distributed random variables $\left\{ {S_{n} } \right\}$, $Y$ and vector $Y^{\prime}$ belong to one independent identically distributed random variables $\left\{ {W_{n} } \right\}$, and $\Delta_{na} > \Delta_{nb}$, then we got $\Delta_{ma} > \Delta_{mb}$. The details of mathematical explanations of the two conclusions for a single sample are given in Note S1. For convenience, if $\Delta_{na} > \Delta_{nb}$ (or $\Delta_{na} < \Delta_{nb}$) in reference*1*, and $\Delta_{ma} > \Delta_{mb}$ (or $\Delta_{ma} < \Delta_{mb}$) in reference*2*, that means, $\Delta_{na}$ and $\Delta_{nb}$, $\Delta_{ma}$ and $\Delta_{mb}$ have the same relationship, we defined it as single-sample-Pearson correlation coefficients, which implies as *s-PCC* in the following paper*.*

### Constructing an individual-specific differential network

**Fig. 1:**
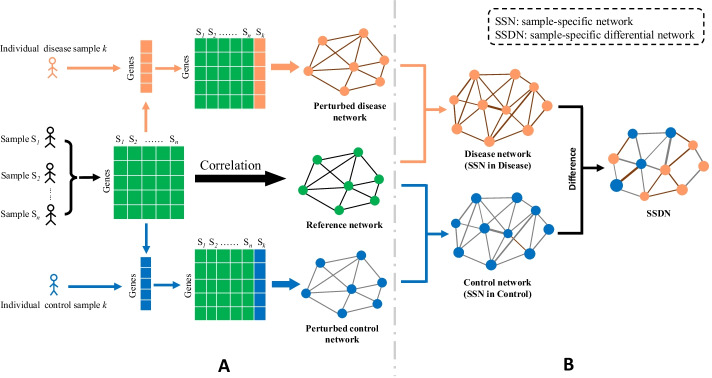
Construction of individual-specific different network. **A** A reference network is established through a set of control samples by *PCC*. A patient sample *k* includes a paired tumor sample (disease sample) and an adjacent normal tissues sample (control sample). A disease sample *k* is added to the reference network and constructed a Perturbed disease network (SSN in Disease). A Perturbed control network (SSN in Control) is obtained by a control sample *k* in the same way. The SSN is a sample-specific network. **B** Based on the theory of predecessors, the Disease network and Control network is constituted by the significance of each edge. The Sample-Specific Differential Network (SSDN) is constructed by quantifying above two networks’ differences

The sample-specific network for an individual patient is constructed based on the statistical perturbation analysis of this sample against a group of given control samples. So, we required expression profiles for a group of normal samples, which served as the reference/control samples. We construct a reference network by Pearson correlation coefficients (*PCC*) using the reference samples (Fig. 1A). We calculate the *PCC* of each pair of genes as an edge with or without a background [24]. Then, a disease sample *k* obtained from cancer tissues of a patient was added to the reference samples and construct a Perturbed disease network by *PCC* (Fig. 1A). After that, a Disease network for disease sample *k* can be obtained by calculating the different edges between the Perturbed disease network and Reference network, and a Disease network is an SSN for the disease sample *k* in disease status (Fig. 1B). At the same time, we also add a control sample *k*, which was obtained from normal tissue of the same patient to the reference network to construct the perturbed control network (Fig. 1A) and Control network (Fig. 1B) through the same procedure. The Control network is an SSN from a control sample. The disease sample *k* was from the tumor tissue of patient *k*, and the control sample *k* was from the tumor-adjacent tissue of patient *k*. The difference between the Disease network and Control network is probably due to cancer-related genes. If the changes between two networks in terms of the network structure are obvious, the genes that caused the changes are highly possible to be cancer-related. On the contrary, if genes are insignificantly changing in the structure between two networks, these are likely not to be cancer-related. Thus, this new network was called sample-specific differential network (SSDN) for sample *k* by obtaining the differences between the two networks (Fig. 1B), i.e. for an edge in the Disease network, if it is not in the Control network, then the edge was kept in final SSDN, and vice versa.

In this study, a protein–protein interaction network (PPIN) from the HPRD database (Human Protein Reference Database, http://www.hprd.org/) was used as the background network to filter the potential false-positive edges from the correlation networks. If there is an edge in PPIN for a pair of genes, the *PCC* of the gene pair would be calculated for the Reference network, Perturbed disease network, and Perturbed normal network. If there is no connection in PPIN for a pair of genes, we ignored the calculation of *PCC* for the gene pair. We used the background network to reduce the amount of calculation of the *PCC* and reduce the existence of false-positive gene pairs (Note S2).

## Results

### Numerical simulation of s-PCC based on different reference networks

To verify our conclusion, a numerical simulation was done for a single sample correlation. In the above paper, we have proved that *PCC* is consistent in two cases, and defined the single-sample-Pearson correlation coefficients as *s-PCC.* Firstly, two simulated sample sets were generated based on normal distributions from the different mean, variance, and sample size as two reference datasets, and the two reference datasets were called reference*1* and reference*2*. Secondly, the *s-PCC* between two genes can be calculated based on the two reference datasets (reference*1* and reference*2*), to obtain *s-PCC1* and *s-PCC2* in a simulated single sample. Assuming the gene-pair $\left( {x,y} \right)$ and $\left( {x^{\prime } ,y^{\prime } } \right)$ in the single sample, the correlations of the two gene-pairs based on reference*1* are *s-PCC1*_*(x, y)*_ and *s-PCC1*_*(x’, y’)*_, the two gene-pairs based on reference*2* are *s-PCC2*_*(x, y)*_, and *s-PCC2*_*(x’, y’)*_. If *s-PCC1*_*(x, y)*_ > *s-PCC1*_*(x’, y’)*_ and *s-PCC2*_*(x, y)*_ > *s-PCC2*_*(x’, y’)*_, or *s-PCC1*_*(x, y)*_ < *s-PCC1*_*(x’, y’)*_ and *s-PCC2*_*(x, y)*_ < *s-PCC2*_*(x’, y’)*_, means the tendency of the *s-PCC* for the two gene-pairs is consistently based on the two reference datasets. We regarded the two gene-pairs as consistent gene-pairs. Then the consistency of *s-PCC* for two reference datasets was defined as the percentage of consistent gene-pairs between the two reference datasets. Finally, we evaluate the consistency of *s-PCC* among different reference datasets.

**Fig. 2:**
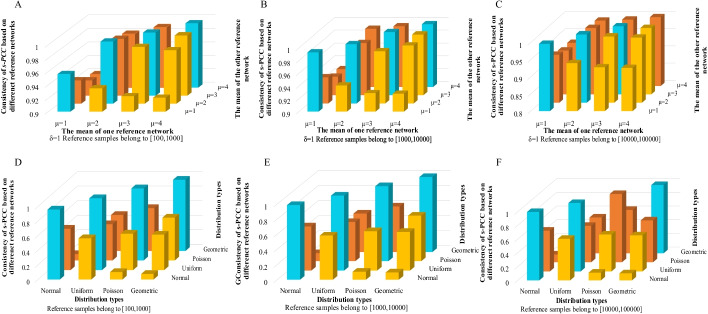
Simulation data verify our conclusions. **A** The variance of two normal distributions is one. Two reference datasets ranging from 100 to 1000. **B** The variance of two normal distributions is one. Two reference datasets ranging from 1000 to 10,000. **C** The variance of two normal distributions is one. Two reference datasets ranging from 10,000 to 100,000. **D** Randomly generated four distributions to verify our conclusion. Two reference datasets ranging from 100 to 1000. **E** Randomly generated four distributions to verify our conclusion. Two reference datasets ranging from 1000 to 10,000. **F** Randomly generated four distributions to verify our conclusion. Two reference datasets ranging from 10,000 to 100,000

**Fig. 3:**
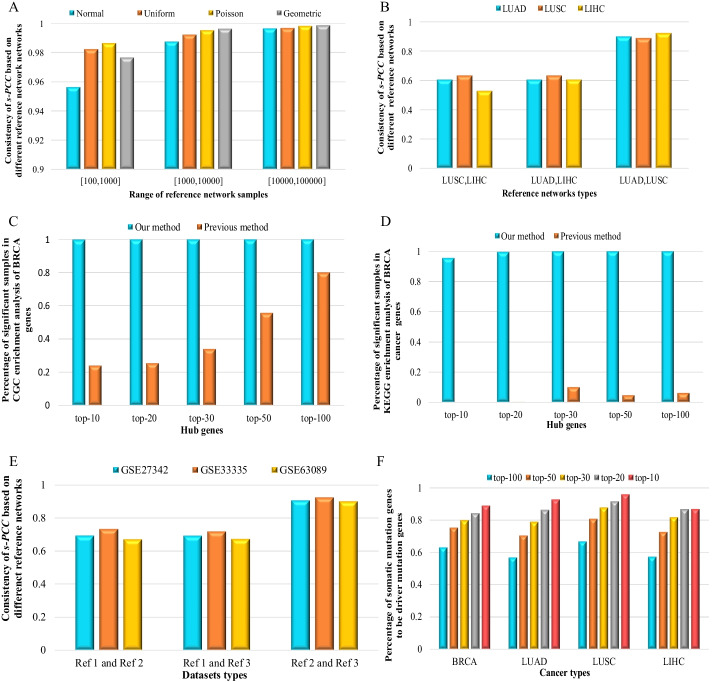
Validating sample-individual differential networks and predicting driver genes in cancer. **A** Four distribution samples ranging from 100 to 1000, 1000 to 10,000 and 10,000 to 100,000. **B** Cross validation of three cancer. **C** Cross validation of three gastric cancer databases. **D** The proportion of significant samples in the enrichment analysis of top 100, 50, 30, 20 and 10 highest degree genes for BRCA DSSN in the KEGG pathway and compare with previous method (SSN). **E** The proportion of significant samples in the enrichment analysis of top 100, 50, 30, 20 and 10 highest degree genes for BRCA DSSN in the CGC database and compare with previous method (SSN). **F** The proportion of somatic mutation genes to be driver mutation genes in top 100, 50, 30, 20 and 10 highest degree genes for each DSSN

The two reference datasets were respectively generated from the normal distribution with the mean value (μ = 1, 2, 3, 4) and the variance (δ = 1), and the sample size of the two reference datasets were the same and randomly obtained from a range. The consistency of *s-PCC* based on different sample sizes of reference dataset was shown in Fig. 2A–C (random value from range 100 to 1000, range 1000 to 10,000, and range 10,000 to 100,000). If two reference datasets were generated from the same distribution (same mean and variance), the consistency of *s-PCC* would be higher than in other situations that the two reference datasets came from different distributions (different mean) (Fig. 2A–C). For example, the two reference datasets range from 100 to 1000, and are generated from the same normal distribution with same mean (μ = 1) and variance (δ = 1), the consistency of *s-PCC* is 95.64% (Fig. 2A). When the two reference datasets generated from different distributions with different mean (μ = 1 and μ = 2) and same variance (δ = 1), the consistency of *s-PCC* is 93.51% (Fig. 2A). When the two reference datasets generated from the distributions μ = 1 and μ = 3 and same variance (δ = 1), the consistency of *s-PCC* is 92.33% (Fig. 2A). When the two reference datasets generated from the distributions μ = 1 and μ = 4 and same variance (δ = 1), the consistency of *s-PCC* is 92.1% (Fig. 2A). The more different the distributions of the two reference datasets generate from, the lower consistency of *s-PCC* for the two reference datasets is. The same tendency was also shown in Fig. 2B, C. And with the increase of sample size of reference datasets, the consistency of *s-PCC* was also raised from range 100 to 1000, range 1000 to 10,000, and range 10,000 to 100,000 (Fig. 2A–C). If two reference datasets generated from the different distributions (Normal Distribution, Uniform Distribution, Poisson Distribution, Geometric Distribution), a similar tendency was also shown in Fig. 2D–F. For example, the two reference datasets range from 100 to 1000, when the reference datasets both generated from Normal Distributions, the consistency of *s-PCC* is 95.58% (Fig. 2D). If one reference dataset generated from Normal Distribution, the other generated from Uniform Distribution, the consistency of *s-PCC* is 56.23% (Fig. 2D). If one reference dataset generated from Normal Distribution, the other generated from Poisson Distribution, the consistency of *s-PCC* is 10.23% (Fig. 2D). If one reference datasets generated from Normal Distribution, the other generated from Geometric Distribution, the consistency of *s-PCC* is 7.65% (Fig. 2D). The same tendency was also shown in Fig. 2E, F. If two reference datasets both generated from Normal Distributions, with the increase of sample size of reference datasets, the consistency of *s-PCC* was also raised from range 100 to 1000, range 1000 to 10,000, and range 10,000 to 100,000 (Fig. 3A). The results of numerical simulation showed that the consistency of *s-PCC* would reduce with the different distribution of the reference datasets, and raise with the increase of sample size of the reference datasets. It is also consistent with the theoretical analysis in the last section.

### Real data validation for the consistency of s-PCC in different reference sets

In addition to the simulated data, three tumor datasets (LUAD, LUSC, and LIHC) were obtained from TCGA (https://www.cancer.gov/) database to validate the results. The control/normal samples (more than ten samples) were randomly selected from the three datasets to form three reference sample sets, and used to construct three reference networks. Each tumor sample in the three tumor datasets constructed Perturbed disease networks based on the three reference networks. The average consistency of *s-PCC* was calculated based on different reference networks, and the random selection from Perturbed disease networks for different gene pairs was repeated ${10}^{5}$ times. We regarded that the normal samples from the same tissue follow the same distributions, so the normal samples from Lung adenocarcinoma (LUAD) and Lung squamous cell carcinoma (LUSC) follow the same distribution, and Liver Hepatocellular Carcinoma (LIHC) follows other distribution compare with LUAD and LUSC. The results showed that if we only used the LUAD dataset as reference sample sets to construct reference networks, the average consistency of *s-PCC* is 91.8%. If we only used the LUSC dataset as reference sample sets to construct reference networks, the average consistency of *s-PCC* is 92%. If we only used the LIHC dataset as reference sample sets to construct reference networks, the average consistency of *s-PCC* is 92.6%. If we used the reference sample sets from LUAD and LUSC, the average consistency is 90.4% (Fig. 3B). While changing the reference sample sets to LUAD and LIHC, the average consistency is changed to 61.61% (Fig. 3B). The average consistency is 60.66% when the reference sample sets were LUSC and LIHC (Fig. 3B). The results showed when the reference networks obey the same distribution, the consistency of *s-PCC* will be higher than the reference networks with a different distribution (Fig. 3B).

Here we also used three gastric cancer databases from the GEO database (https://www.ncbi.nlm.nih.gov/geo/) as an example, these datasets are GSE33335, GSE63089, and GSE27342. We selected the control/normal samples of the three datasets as the reference samples to construct three reference networks, and each tumor sample was used to construct Perturbed disease networks based on the three reference networks. For convenience, we noted the reference network from GSE33335 as *Ref1*, from GSE63089 as *Ref2* and from GSE27342 as *Ref3*. The number of reference samples in GSE33335 was 24, GSE63089 was 45, GSE27342 was 80. The average consistency of *s-PCC* was calculated based on different reference networks and the random selection from Perturbed disease networks for different gene pairs was repeated ${10}^{5}$ times. The results were shown that if taken *Ref1* and *Ref2* as reference networks, constructed the Perturbed disease networks for GSE27342, the consistency of *s-PCC* was 69.34%. constructed the Perturbed disease networks for GSE 33,335, the consistency of *s-PCC* was 73.28%, constructed the Perturbed disease networks for GSE63089, the consistency of *s-PCC* was 67.18% (Table 1 and Fig. 3C). When the Perturbed disease networks were constructed taken *Ref1* and *Ref3* as reference network, the consistency of the three datasets was similar to the consistency based on *Ref1* and *Ref2* (Table 1 and Fig. 3C). And when the Perturbed disease networks were constructed taken *Ref2* and *Ref3* as reference network, the consistency would be rapidly increased by over 90% (Table 1 and Fig. 3C). It is an agreement with the theoretical derivation that the consistency of *s-PCC* would be raised with the increase number of reference samples.

**Table 1:** The comparison for the consistency of different reference samples

Dataset	*Ref1* and *Ref2*	*Ref1* and *Ref3*	*Ref2* and *Ref3*
GSE27342	69.34%	69.23%	90.71%
GSE33335	73.28%	71.80%	92.55%
GSE63089	67.18%	67.35%	90.20%

### DSSN reveal individual features by pathway and disease gene enrichment

For revealing the disease modules of non-paired tumor samples, a common normal network was constructed by collecting the common edges of Control networks, the edges existed in more than 1/3 Control networks for a type of cancer. The common normal network was used as the Control network to deduce the patient-specific disease modules. A disease-specific sample network (DSSN) was established by identifying the differential edges between the Disease network and the Control network (Fig. 1). That is, for an edge in the Disease network, if it is not in the control network, then the edge was kept in the final DSSN. The hub nodes of DSSN were the potential cause modules of this tumor sample, and then the top- 100, 50, 30, 20 and 10 hub genes with a high degree in DSSN were respectively selected as potential disease modules for every tumor sample in TCGA.

**Fig. 4:**
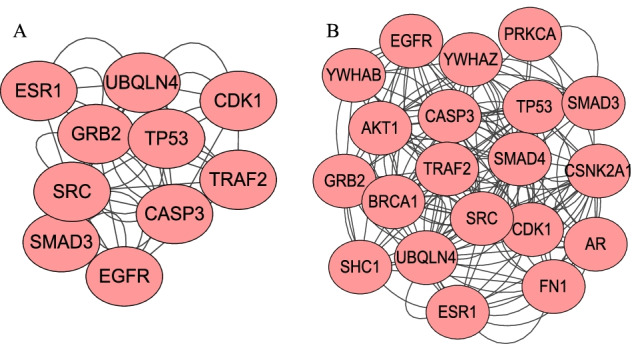
The potential disease modules in BRCA reference network. **A** The network modules among the top- 10 hub genes. **B** The network modules among the top- 20 hub genes

**Fig. 5:**
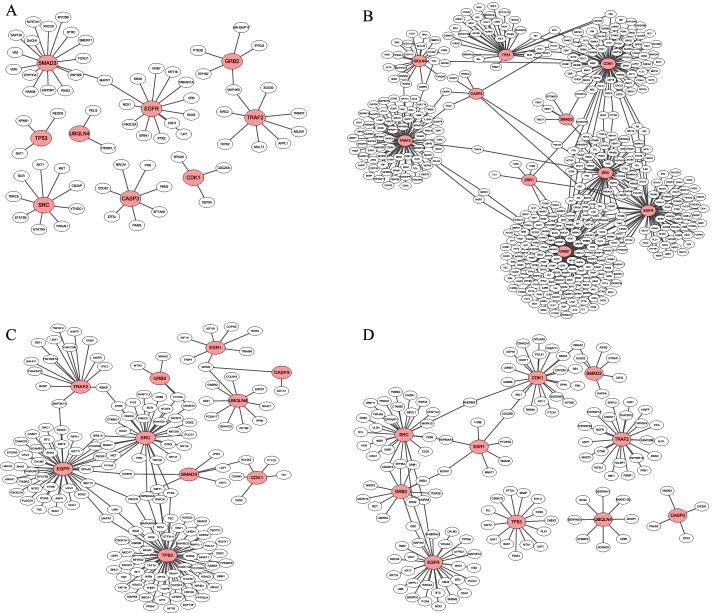
The potential disease modules in BRCA Control network and Disease network. **A** The network modules among the top- 10 hub gene in Control network. **B** The network modules among the top- 10 hub gene in Disease network in sample BRCA_AAAK. **C** The network modules among the top- 10 hub gene in Disease network in sample BRCA_A0CZ. **D** The network modules among the top- 10 hub gene in Disease network in sample BRCA_A440

We chose the Breast invasive carcinoma (BRCA) and LIHC to draw the disease modules networks. Disease modules refelect diffrerent extent of aggregation in different networks. In BRCA reference network, we selected the top- 10 and 20 hub genes as potential disease modules, and calculated the *PCC* between these genes. The gene pairs with p-value less than 0.01 form the edges of the network (Fig. 4). In BRCA Control network, the top- 10 disease modules cannot be aggregated, while are scattered by several modules (Fig. 5A). Because our modules are selected from Disease network hub genes, so it cannot be significantly aggregated in the Control network. In BRCA Disease network, we selected three individual samples to view the network (Fig. 5B–D). The top- 10 disease modules are significantly aggregated in the Disease network. The top 20- disease modules in Disease network are also shown in Additional file 9: Figure S1. In the same way, the top- 10 and 20 disease modules in LIHC are drawn in Additional files 10–12: Figure S2-S4. It also implies that hub genes exited in the form of modules in the Disease network.

The potential disease modules of each sample were enriched to the corresponding pathway in KEGG and enriched the disease genes in CGC database. The hypergeometric test was used to test the significant level of the enrichment analysis and the percentage of significant samples. The results showed that the top- 100, 50, 30, 20 and 10 hub genes of more than 95% samples in BRCA were significantly enriched to Pathways in cancer and Breast cancer pathway in KEGG (Fig. 3D, Table 2). All potential disease modules (top- 100, 50, 30,20 and 10 hub genes) were significantly enriched to tumor genes in CGC database (Fig. 3E, Table 3). There are similar results for the potential disease modules in LUAD, LUSC, and LIHC with BRCA (Additional file 13: Figure S5). For example, there are more than 95% LIHC samples to be significantly enriched to Pathways in cancer and Hepatocellular carcinoma pathway in KEGG and all samples to be significantly enriched to existing tumor genes in CGC by top-100, 50, 30, 20 and 10 genes of DSSN (Additional file 13: Figure S5C and F). For LUAD and LUSC, the potential disease modules of almost all tumor samples were significantly enriched corresponding Pathways in cancer, Non-small cell lung cancer pathway in KEGG and existing tumor genes in CGC (Additional file 13: Figure S5A, B, D, and E). Compared with the SSN method [24], our method can obtain higher accuracy of significant samples in the enrichment analysis of disease pathways and cancer-related genes by the potential disease modules from DSSN.

**Table 2:** A comparison of the KEGG enrichment analyses between our method and the SSN method

BRCA	Top-10	Top-20	Top-30	Top-50	Top-100
Our method	95.64%	99.64%	99.99%	100%	100%
SSN method	0.00%	0.18%	10.00%	4.63%	6.17%
LUAD	top-10	top-20	top-30	top-50	top-100
Our method	96.81%	95.68%	99.81%	100%	100%
SSN method	0.00%	0.00%	0.38%	3.57%	15.57%
LUSC	top-10	top-20	top-30	top-50	top-100
Our method	98.41%	97.21%	99.20%	100%	100%
SSN method	0.20%	0.00%	0.80%	4.38%	12.25%
LIHC	top-10	top-20	top-30	top-50	top-100
Our method	100.00%	100%	100%	100%	100%
SSN method	0.00%	0.00%	0.54%	3.78%	7.55%

**Table 3:** A comparison of the CGC enrichment analyses between our method and the SSN method

BRCA	top-10	top-20	top-30	top-50	top-100
Our method	99.82%	100.00%	100.00%	100.00%	100.00%
SSN method	23.96%	25.41%	34.03%	55.63%	80.03%
LUAD	top-10	top-20	top-30	top-50	top-100
Our method	99.62%	100.00%	100.00%	100.00%	100.00%
SSN method	16.89%	22.14%	24.39%	50.09%	76.55%
LUSC	top-10	top-20	top-30	top-50	top-100
Our method	100.00%	100.00%	100.00%	100.00%	100.00%
SSN method	7.37%	10.76%	12.95%	32.07%	69.52%
LIHC	top-10	top-20	top-30	top-50	top-100
Our method	98.92%	99.73%	100.00%	100.00%	100.00%
SSN method	10.78%	10.51%	15.36%	30.19%	60.91%

### Predicting individual driver mutation by DSSN

Somatic mutation genes of a tumor sample can provide individual-specific information for this sample [28] and can be used to verify the potential driver genes of the sample. There are 125 existing driver mutation genes to have been determined for cancer in reference [29]. As we have referred, a hub gene in SSDN is a crucial gene from normal to tumor state. If a hub gene of SSDN was mutated, the gene may impact more genes than the non-hub gene and would be the potential driver mutation gene for this sample. Based on such an assumption, DSSN was involved in the network change between normal and tumor, and the hub genes in DSSN are more likely to associate with disease. So, the probability/proportion, which a mutated gene is an existing driver mutation gene, was respectively calculated for the top-100, 50, 30, 20 and 10 hub genes of each DSSN in each tumor and the average probability of each tumor was shown in Fig. 3F. The results showed that the probability/proportion was monotonically increased from the top- 100 to 10 hub genes of DSSN (Fig. 3F). As an example, if a gene in the top 100 hub genes of DSSN of a BRCA sample was mutated, the probability of this gene being a driver mutation gene is 63.04% (Fig. 3F). If a gene was mutated in the top 10 hub genes of DSSN of a BRCA sample, the probability would rise to 88.96% (Fig. 3F). It means if a hub gene of DSSN is mutated, the gene is a high probability to be a driver mutation gene in BRCA. Similar results were shown in LUAD, LUSC, and LIHC (Fig. 3F). Therefore, the hub genes of DSSN are strongly related to the disease cause for one sample, and high-degree genes are more likely to be carcinogenic factors.

### Prognosis analysis for tumor samples

The clinic follow-up information was collected for each sample in TCGA, and the survival times (unit is days) and vital status (alive or dead) were filtered out. The samples that missed survival times or vital status were ignored. For BRCA and LIHC, the repetition hub genes were identified based on the top 10 hub genes of each DSSN, and the most frequent 10 repetition hub genes were used to survival analysis for tumor samples. We used the 10 repetition hub genes to divide tumor samples into two groups, one included the samples that there were at least 4 repetition hub genes to be in the top 10 hub genes of this sample; another included the samples that had less than 4 repetition hub genes to be in the top 10 hub genes of this sample. The repetition hub genes can also be identified based on the top 20 hub genes of each DSSN, and the most frequent 20 repetition hub genes were used to survival analysis for tumor samples. In the same way, the 20 repetition hub genes to divide tumor samples into two groups, one included the samples that there were at least 9 repetition hub genes to be in the top 20 hub genes of this sample; another included the samples that had less than 9 repetition hub genes to be in the top 20 hub genes of this sample. A log-rank test was employed to test the significance of the survival time.

**Fig. 6:**
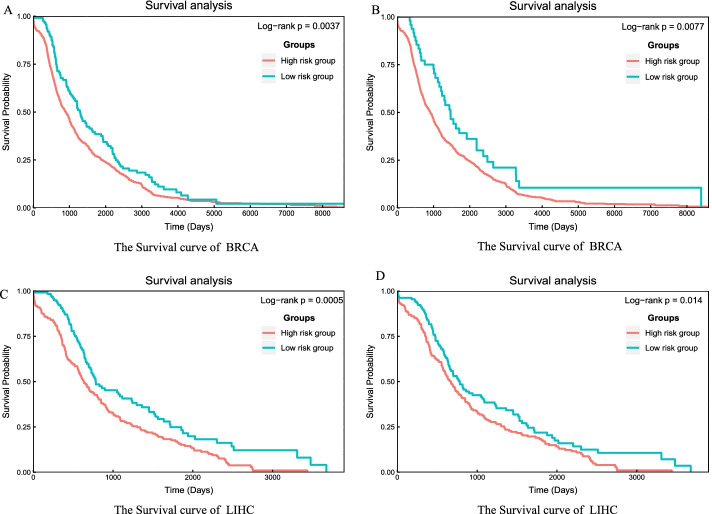
Survival curve for BRCA and LIHC. **A** Survival curve for BRCA survival analysis when using the most frequent 10 repetition hub genes to divide tumor samples into two groups. **B** Survival curve for BRCA survival analysis when using the most frequent 20 repetition hub genes to divide tumor samples into two groups. **C** Survival curve for LIHC survival analysis when using the most frequent 10 repetition hub genes to divide tumor samples into two groups. **D** Survival curve for LIHC survival analysis when using the most frequent 20 repetition hub genes to divide tumor samples into two groups

**Fig. 7:**
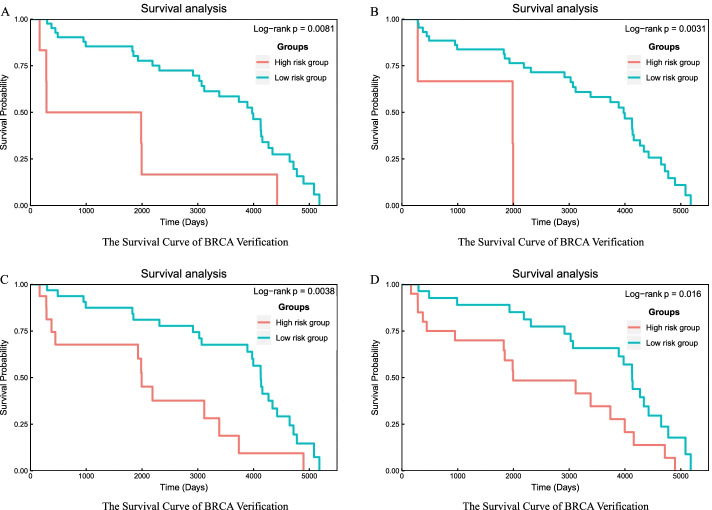
Examine the statistical significance of hub genes using BRCA test samples. **A** Survival curve for BRCA verification survival analysis when using the most frequent 10 repetition hub genes to divide tumor samples into two groups. **B** Survival curve for BRCA verification survival analysis when using the most frequent 20 repetition hub genes to divide tumor samples into two groups. **C** Survival curve for BRCA verification survival analysis when using the most frequent 30 repetition hub genes to divide tumor samples into two groups. **D** Survival curve for BRCA verification survival analysis when using the most frequent 50 repetition hub genes to divide tumor samples into two groups

The most frequent 10 and 20 repetition hub genes can significantly distinguish the samples with different survival time. For BRCA, the most frequent 10 repetition hub genes samples in the two groups (high and low risk) can be significantly distinguished with *p*-value 0.0037 of log-rank test (Fig. 6A); the most frequent 20 repetition hub genes samples in the two groups also can be significantly distinguished with *p*-value 0.0077 of log-rank test (Fig. 6B). The *p*-value of the most frequent 10 and 20 repetition hub genes of LIHC samples for survival analysis are respectively 0.0005 and 0.014 (Fig. 6C, D). Including LUSC samples, the repetition hub genes can be used to prognosis analysis in BRCA, LUAD, and LIHC (Fig. 6, Additional file 14: Figure S6). An independent dataset from ICGC database for BRCA was used to validate the effectiveness of the DSSN in survival analysis, and the significant results were shown in Fig. 7. The repetition hub genes can be considered as the potential biomarkers for prognosis of the tumors.

## Conclusion

In this work, we proposed the SSDN method and overcame the shortcomings of the previous method. We compared the difference between the SSN in Disease and SSN in Control, constructed the SSDN and DSSN. And we verified the consistency of *s-PCC* based on different reference networks from both theoretical and practical. SSDN and DSSN can also be used to select disease modules (hub genes) to evaluate individual features. The enrichment analysis of KEGG pathway and CGC database indicated the disease modules play an important role in cancer-related function by using the TCGA datasets for various cancer. The results of survival analysis demonstrated these genes can be used independently as individual module biomarkers. We expect SSDN and DSSN to generalize well to further studies, including application to pathological diagnosis and drug therapy for patient-specific cancer care.

## Discussion

The SSDN method is an improvement version of SSN method. The SSN method uses the reference network, but it does not explain whether the choice of different reference networks will affect the construction of Disease/Control network. We used the mathematical explanations to prove that in two cases, one is the size of samples is sufficiently large, other is the samples come from the same distribution. We have proved that the *PCC* in these two cases are consistent. And verified that the different reference networks have no effect on the construction of the network by using the real cancer data. Another improvement is the SSN method have constructed the individual-specific network for disease sample and is called the Disease network, the individual-specific network for control sample is called the Control network. We compared the two networks, and found the difference between the Disease network and Control network. Because if we only rely on the *PCC* to judge the significance of a gene pair/edge, there will have a high false positive. Comparing the difference between the two networks, we can select the gene pairs that only exist in the Disease network, or select the gene pairs that only exist in the Control network. In this way, we can reduce the false positive gene pairs, find the real difference between the two networks and remove the disturbance. Moreover, by using the real cancer data, we verified the potential disease modules for individual sample in SSDN method were enriched to the corresponding pathway in KEGG and enriched to the disease genes in CGC database (Tables 2, 3).

Several limitations of the current study should be considered, based on the limitations, we construct two kinds of different networks, SSDN and DSSN. For constructing SSDN, a sample pair are necessary for every patient. It means that there must be a tumor and control sample from the same patient tissue, respectively, only then we can construct a Disease network and a Control network for each patient. While if the normal samples cannot correspond to tumor samples, the situation has changed slightly. We create a new network, common normal network, for which the edge includes more than 1/3 Control networks’ edges for a same cancer. The common normal network was called the Control network, and the rest steps of constructing DSSN are the same as SSDN. For an edge in the Disease network, if it is not in the Control network, then the edge was kept in the DSSN. The DSSN is a disease-specific sample network because the common normal network is constructed based on the same cancer, the subsequent conclusions are also concluded on the same cancer. Therefore, SSDN is constructed by identifying the different edges between the SSN in disease and SSN in control, DSSN is constructed by identifying the different edges between the SSN in disease and common normal network. Here, in order to construct SSDN, we used three gastric cancer datasets from the GEO database because the samples with the same cancer obey the same distribution. These three datasets have 25, 45, and 80 paired samples, which include tumor and control samples from the same patient tissue. Since paired samples are two samples from the same patient, we can construct a Disease network and a Control network for individual patient. However, by analyzing four cancer datasets from TCGA database, due to the normal samples that cannot correspond to tumor samples, we have to use DSSN to solve the problem. Although DSSN cannot completely consistent with our previous methods, it indeed the best way to solve the problem of missing data.

We used three gastric cancer datasets from the GEO database and these datasets are GSE33335, GSE63089, and GSE27342. For convenience, we noted the reference network from GSE33335 as *Ref1*, from GSE63089 as *Ref2* and from GSE27342 as *Ref3*. We used the *Ref2* and *Ref3* as two reference networks. For example, we add a normal sample of GSE27342 in a reference network and obtain the *s-PCC* between each gene pair. If the relationship between a pair of genes is significant, this pair of genes can be linked to an edge in the Control network. In the same way, we add a disease sample of GSE27342 in reference networks and obtain a Disease network. Hence, there is a Disease network and a Control network for a individual sample. For the Disease network and Control network, there are six ways to obtain SSDN. The first SSDN is constructed based on specific genes in Disease and Control networks, the second SSDN is constructed based on common genes in Disease and Control network, and the rest are constructed based on the specific genes in Control networks, the specific genes in Disease networks, the genes only in Control networks, and the genes only in Disease networks. We selected the most frequent 300, 100, 50, 30, 20 and 10 repetition hub genes, and calculated the percentage of common hub genes based on *Ref1 Ref2*, *Ref3*. For dataset GSE27342 and the most frequent 10 hub genes, the percentage of common hub genes in the first SSDN is 50%, the percentage of common hub genes in the second SSDN is 54.77%. The results of other percentage hub genes were shown in Additional file 16: Table S1. According to our theory and the real data validation, when the sample size is sufficiently large or the distribution is the same, the consistency of *s-PCC* will be very high. While the sample size of the above three gastric cancer datasets is just 25, 45, and 80, the sample size is too small to get a high consistency. The SSDN established based on *Ref2* and *Ref3* with a relatively large sample size is already the best result at present. Furthermore, we hope to develop a method to construct SSDN independent of the sample number.

For the survival analysis, we also used other parameters (the number of top hub genes and the number of repetitions hub genes) to test the robustness of the DSSN. For BRCA, the most frequent 30 repetition hub genes samples in the two groups (high and low risk) can be significantly distinguished with *p*-value 0.013 of the log-rank test (Additional file 10: Figure S2A); the most frequent 50 repetition hub genes samples in the two groups also can be significantly distinguished with *p*-value 0.024 of the log-rank test (Additional file 10: Figure S2A). The results showed that different parameters can obtain similar results for prognosis (Additional file 10: Figure S2).

Compared with the prior work [15], the previous work of Liu, X et al. We took the normal samples of the same cancer as a whole, and calculate the correlation by Liu’s method, and took the disease samples of the same cancer as a whole, calculate the correlation by Liu’s method. Then we calculate the CGC enrichment and KEGG enrichment of the whole cancer. The results show that SSDN method is prior than the Liu’s method. We have proved, although the SSDN method is used for single sample difference analysis, it turns out if treat the cancer as a whole, the enrichment analysis results are still good (Additional file 17: Table S2, Additional file 18: Table S3).

And we compared with the SSN method and our method. For BRCA, we used the SSDN method, and selected the gene pairs that specific existing in the disease network, then identified the top 6 genes with the highest degree in these gene pairs. For one sample, we have constructed the SSN, and calculated the top 10 genes with the highest degree in SSN. If the top 10 genes have less than 2 genes of the top 6 genes, the sample will be regarded as the normal sample. Otherwise, the sample can be regarded as the disease sample. The percentage accuracy of the SSDN classification is 88.56%. On the other hand, we used the SSN method to classify the control sample and disease sample. We have selected the gene pairs that existing in the control network, and calculated the top 6 genes with the highest degree in these gene pairs. Then we have constructed the SSN in control and SSN in disease, and calculated the top 10 genes with the highest degree in SSN. If the top 10 genes have less than 2 genes of the top 6 genes, the sample will be regarded as the control sample, otherwise it’s a disease sample. Finally, we have selected the gene pair that existing in the disease network, and calculated the top 6 genes with the highest degree in these gene pairs. Based on the top 6 genes, we classify again. As a result, for BRCA, the accuracy of SSN in control classification is 54.40%, the accuracy of SSN in disease classification is 85.27%. The accuracy of classification for the other three cancer are also shown in the Additional file 19: Table S4. The result shows that the SSDN method select is indeed the specific gene in the disease network, and is better than SSN method. Because the SSN method can only reflect the information of a single individual, and our method can maximize the use of normal sample and disease sample.

As for survival analysis, we also compared with the SSN method. For BRCA and LIHC, the repetition hub genes were identified based on the top 10 hub genes of each DSSN, and the most frequent 10 repetition hub genes were used to survival analysis for tumor samples. We calculated the top 6 genes with the highest degree in SSN in control, and used the 10 repetition hub genes to divide tumor samples into two groups, one included the samples that there were at least 2 repetition hub genes to be in the top 6 hub genes of this sample; another included the samples that had less than 2 repetition hub genes to be in the top 10 hub genes of this sample. In the same way, we calculate the top 6 genes with the highest degree in SSN in disease, and repeat the same classification process above. The *p*-value in DSSN method is below 0.05 (Fig. 6), is better than SSN (Additional file 20: Table S5). It further illustrates that SSDN/DSSN method can find the difference between disease and control samples.

## Supplementary Information

**Additional file 1**. The cancer gene of BRCA.

**Additional file 2**. The normal gene of BRCA.

**Additional file 3**. The cancer gene of LIHC.

**Additional file 4**. The normal gene of LIHC.

**Additional file 5**. The cancer gene of LUAD.

**Additional file 6**. The normal gene of LUAD.

**Additional file 7**. The cancer gene of LUSC.

**Additional file 8**. The normal gene of  LUSC.

**Additional file 9**. **Figure S1.** The network modules with the potential disease modules in BRCA Control network and Disease network. (A) The network modules among the top- 20 hub gene in Control network. (B) The network modules among the top- 20 hub gene in Disease network in sample BRCA_A0T6. (C) The network modules among the top- 20 hub gene in Disease network in sample BRCA_A4RY. (D) The network modules among the top- 20 hub gene in Disease network in sample BRCA_A1IX.

**Additional file 10**. **Figure S2.** The network modules with the potential disease modules in LIHC reference network. (A) The network modules among the top- 10 hub genes. (B) The network modules among the top- 20 hub genes.

**Additional file 11**. **Figure S3.** The network modules with the potential disease modules in LIHC Control network and Disease network. (A) The network modules among the top- 10 hub gene in Control network. (B) The network modules among the top- 10 hub gene in Disease network in sample LIHC_A9H1. (C) The network modules among the top- 10 hub gene in Disease network in sample LIHC _A69I. (D) The network modules among the top- 10 hub gene in Disease network in sample LIHC _AAC9.

**Additional file 12**. **Figure S4.** The network modules with the potential disease modules in LIHC Control network and Disease network. (A) The network modules among the top- 20 hub gene in Control network. (B) The network modules among the top- 20 hub gene in Disease network in sample LIHC_A110. (C) The network modules among the top- 20 hub gene in Disease network in sample LIHC _A520. (D) The network modules among the top- 20 hub gene in Disease network in sample LIHC _AA0V.

**Additional file 13**. **Figure S5.** The enrichment in KEGG pathway and CGC database compared with our method and SSN method. (A)The proportion of significant samples in the enrichment analysis of top- 100, 50, 30, 20 and 10 highest degree genes for LUAD DSSN in the KEGG pathway and compare with the previous method. The x-axis is the hub genes and the y-axis is the percentage of significant samples in KEGG enrichment analysis. (B) The proportion of significant samples in the enrichment analysis of top- 100, 50, 30, 20 and 10 highest degree genes for LUSC DSSN in the KEGG pathway and compare with the previous method. The x-axis is the hub genes and the y-axis is the percentage of significant samples in KEGG enrichment analysis. (C) The proportion of significant samples in the enrichment analysis of top- 100, 50, 30, 20 and 10 highest degree genes for LIHC DSSN in the KEGG pathway and compare with the previous method. The x-axis is the hub genes and the y-axis is the percentage of significant samples in KEGG enrichment analysis. (D) The proportion of significant samples in the enrichment analysis of top- 100, 50, 30, 20 and 10 highest degree genes for LUAD DSSN in the CGC database and compare with the previous method. The x-axis is the hub genes of cancer and the y-axis is the percentage of significant samples in CGC enrichment analysis. (E) The proportion of significant samples in the enrichment analysis of top- 100, 50, 30, 20 and 10 highest degree genes for LUSC DSSN in the CGC database and compare with the previous method. The x-axis is the hub genes of cancer and the y-axis is the percentage of significant samples in CGC enrichment analysis. (F) The proportion of significant samples in the enrichment analysis of top- 100, 50, 30, 20 and 10 highest degree genes for LIHC DSSN in the CGC database and compare with the previous method. The x-axis is the hub genes of cancer and the y-axis is the percentage of significant samples in CGC enrichment analysis.

**Additional file 14**. **Figure S6.** Survival curve for BRCA and LIHC. (A) Survival curve for BRCA survival analysis when using the most frequent 30 repetition hub genes to divide tumor samples into two groups. (B) Survival curve for BRCA survival analysis when using the most frequent 50 repetition hub genes to divide tumor samples into two groups. (C) Survival curve for LIHC survival analysis when using the most frequent 30 repetition hub genes to divide tumor samples into two groups. (D) Survival curve for LIHC survival analysis when using the most frequent 50 repetition hub genes to divide tumor samples into two groups. (E) Survival curve for LUAD survival analysis when using the most frequent 30 repetition hub genes to divide tumor samples into two groups. (F) Survival curve for LUSC survival analysis when using the most frequent 20 repetition hub genes to divide tumor samples into two groups.

**Additional file 15**. Summary documentation of Supplementary Information.

**Additional file 16**. **Table S1.** For Disease network and Control network, we selected the most repetition 300, 100, 50, 30, 20 and 10 hub genes, compared whether these have common genes under these two reference networks by six ways.

**Additional file 17**. **Table S2.** The enrichment in CGC database compared with our method and previous method.

**Additional file 18**. **Table S3.** The enrichment in KEGG database compared with our method and previous method

**Additional file 19**. **Table S4.** The accuracy of the classification for four cancer.

**Additional file 20**. **Table S5.** Survival analysis for SSN.

## Data Availability

LIHC cancer gene, the raw data of LIHC cancer gene. LIHC normal gene, the raw data of LIHC normal gene. LUSC cancer gene, the raw data of LUSC cancer gene. LUSC normal gene, the raw data of LUSC normal gene. LUAD cancer gene, the raw data of LUAD cancer gene. LUAD normal gene, the raw data of LUAD normal gene. BRCA cancer gene, the raw data of BRCA cancer gene. BRCA normal gene, the raw data of BRCA normal gene. All the datasets generated and analysed during the current study are available in the [TCGA] repository, [https://www.cancer.gov/about-nci/organization/ccg/research/structural-genomics/tcga]. Gastric Cancer Sample, GSE27342 [https://www.ncbi.nlm.nih.gov/geo/query/acc.cgi?acc=GSE27342]. GSE63089 [https://www.ncbi.nlm.nih.gov/geo/query/acc.cgi?acc=GSE63089]. GSE33335 [https://www.ncbi.nlm.nih.gov/geo/query/acc.cgi?acc=GSE33335].
