# Supplementary figures and images for: Identifying network biomarkers of cancer by sample-specific differential network
Source: BMC Bioinformatics. 2022 Jun 15;23:230. doi: 10.1186/s12859-022-04772-1 (PMC9202129; doi:10.1186/s12859-022-04772-1)

A

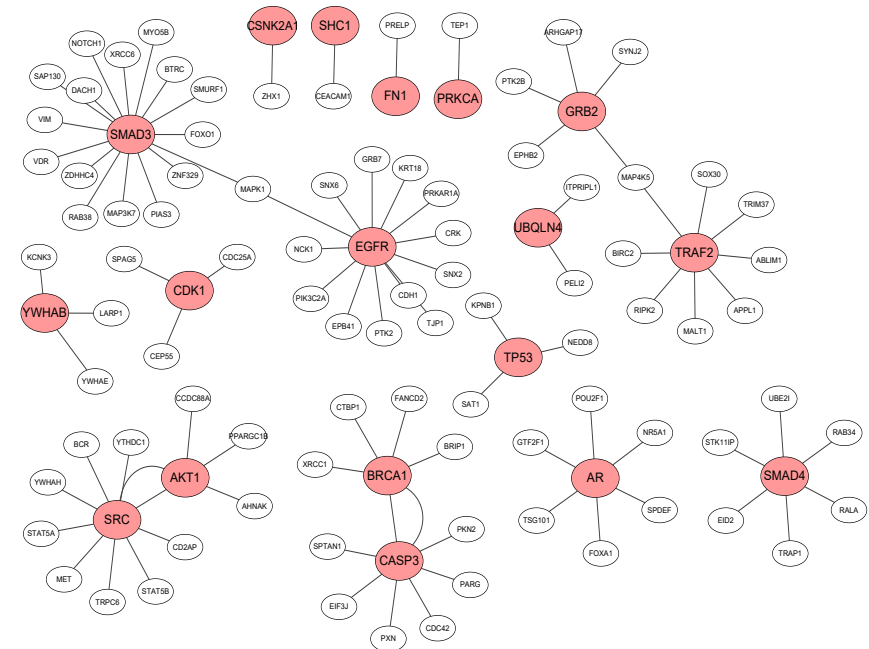

B

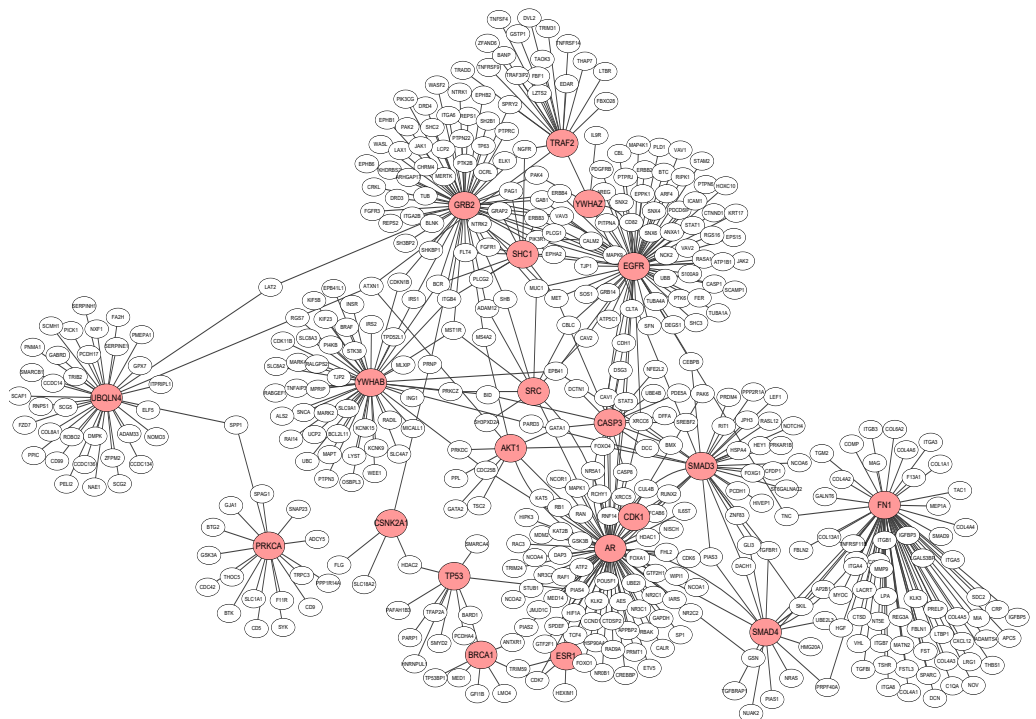

C

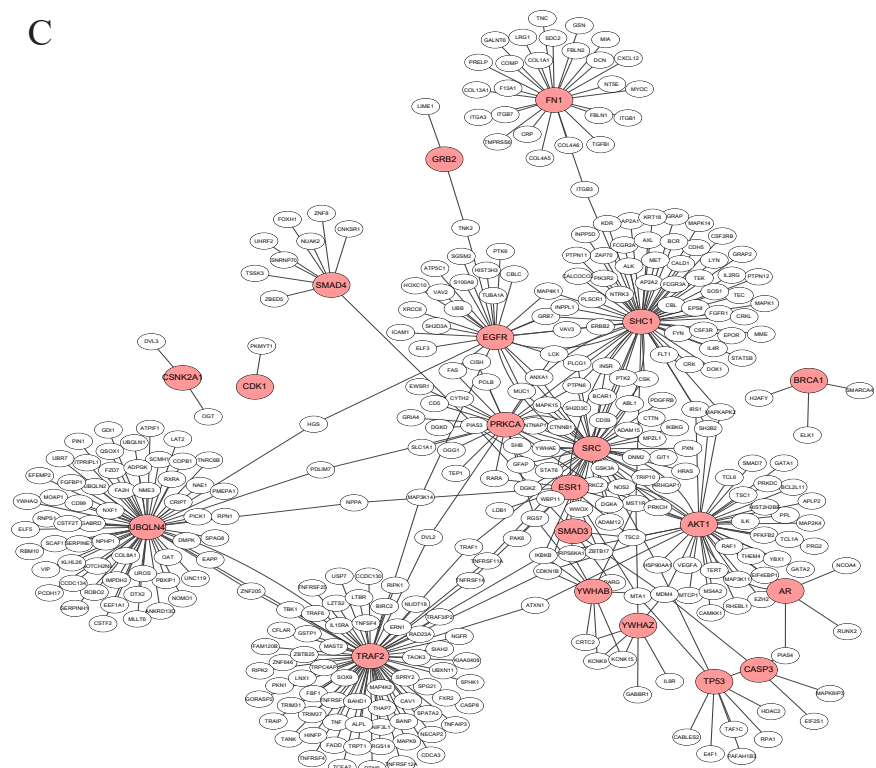

D

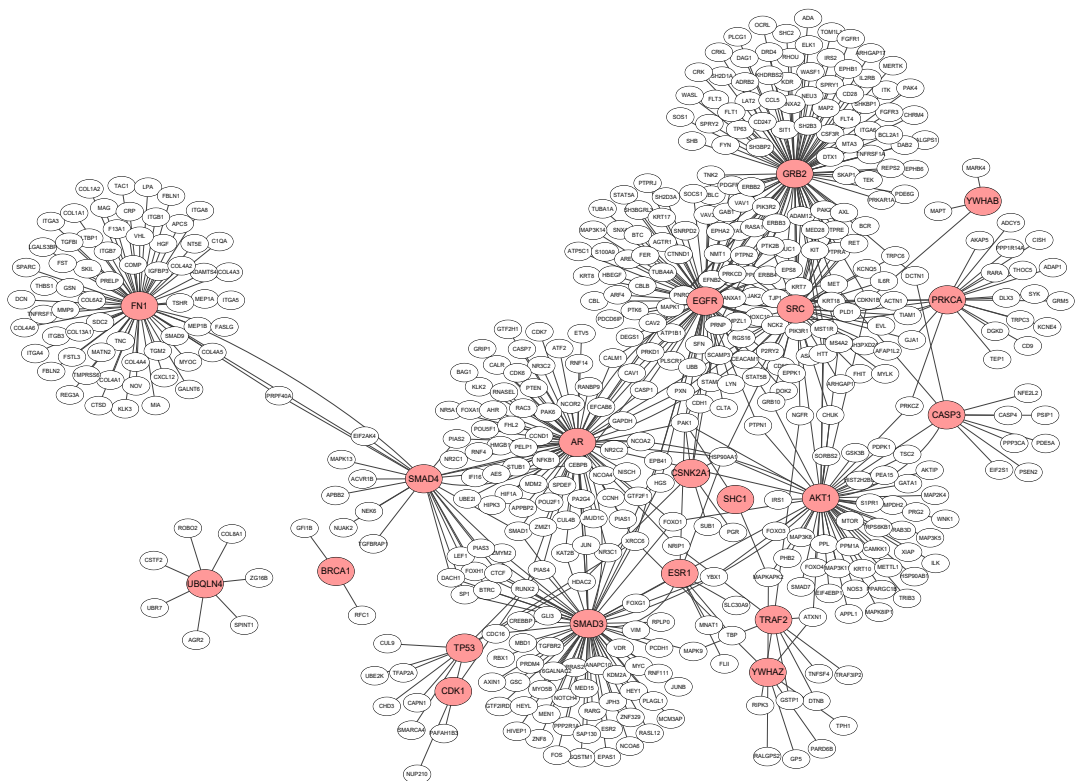

Supplement: Supplementary file 9 — Additional file 9. Figure S1. The network modules with the potential disease modules in BRCA Control network and Disease network. (A) The network modules among the top- 20 hub gene in Control network. (B) The network modules among the top- 20 hub gene in Disease network in sample BRCA_A0T6. (C) The network modules among the top- 20 hub gene in Disease network in sample BRCA_A4RY. (D) The network modules among the top- 20 hub gene in Disease network in sample BRCA_A1IX. [file 12859_2022_4772_MOESM9_ESM.pdf]

A

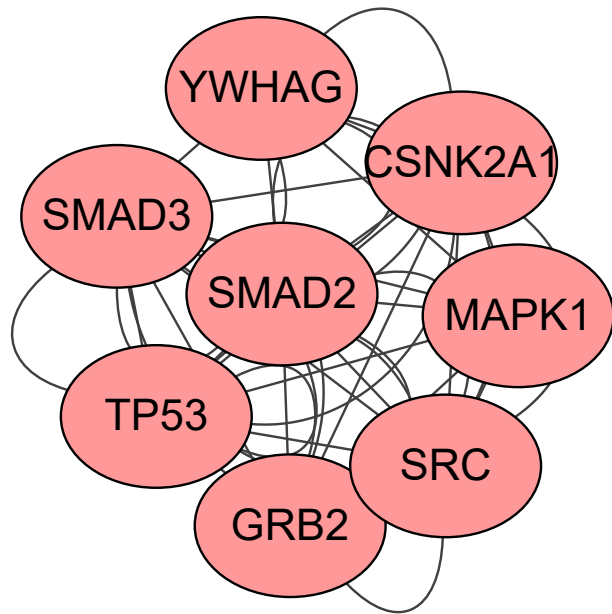

B

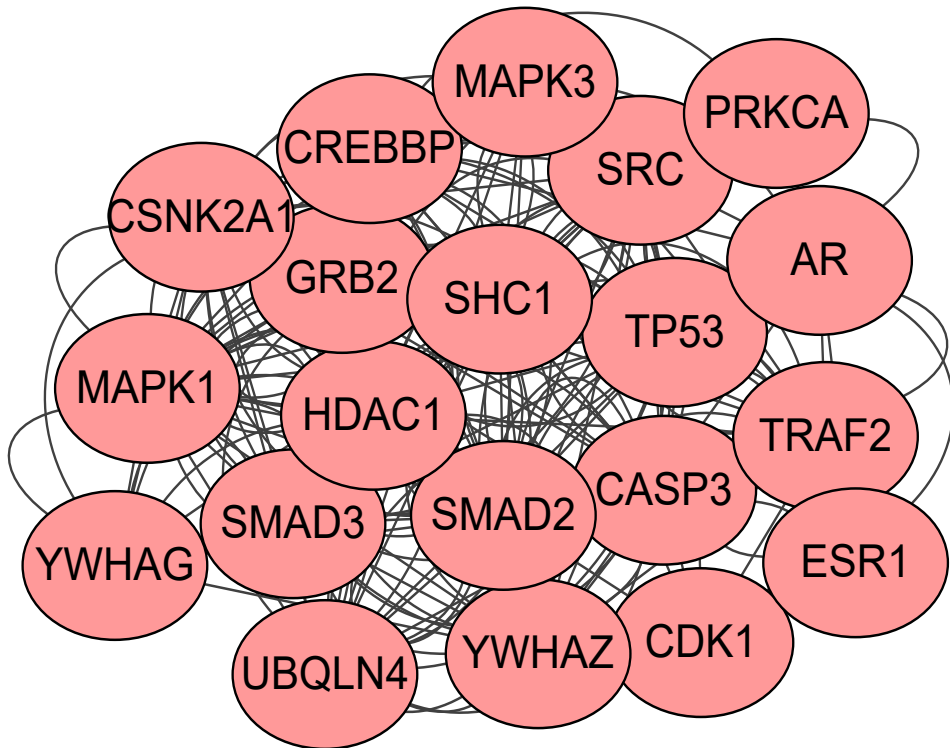

Supplement: Supplementary file 10 — Additional file 10. Figure S2. The network modules with the potential disease modules in LIHC reference network. (A) The network modules among the top- 10 hub genes. (B) The network modules among the top- 20 hub genes. [file 12859_2022_4772_MOESM10_ESM.pdf]

A

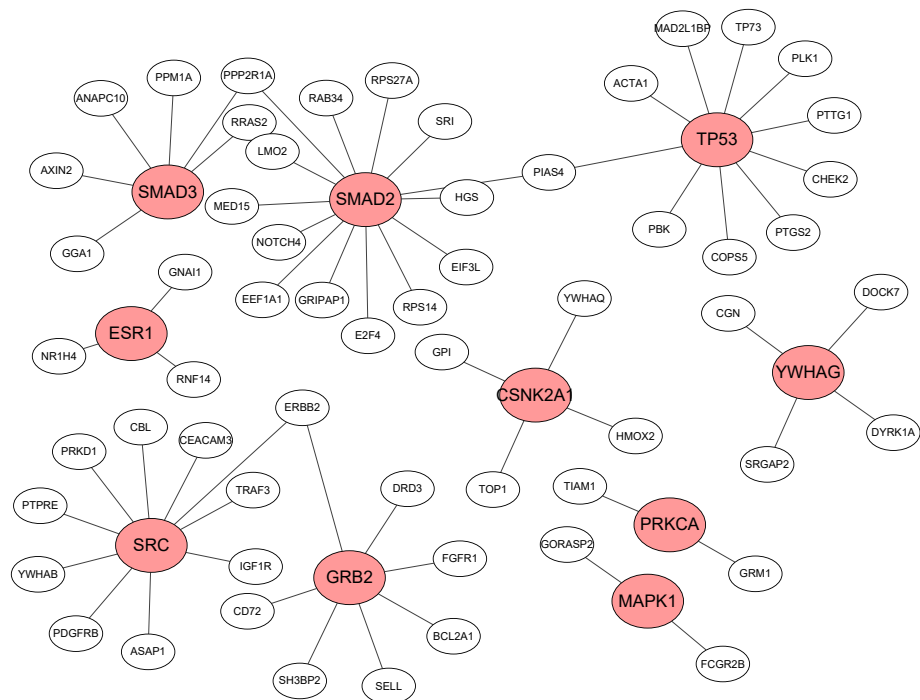

B

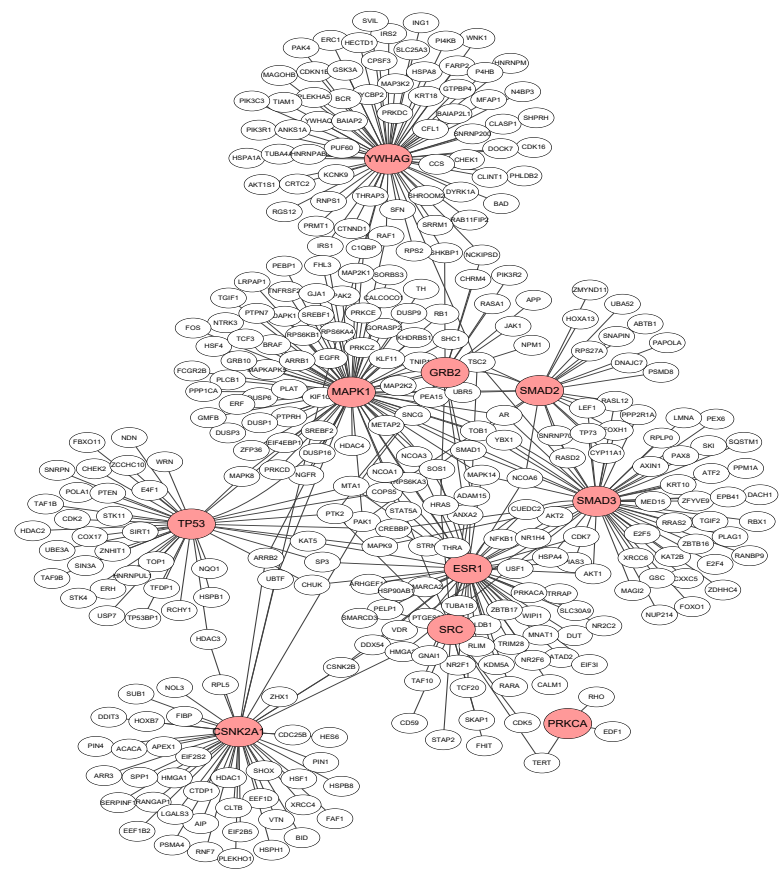

C

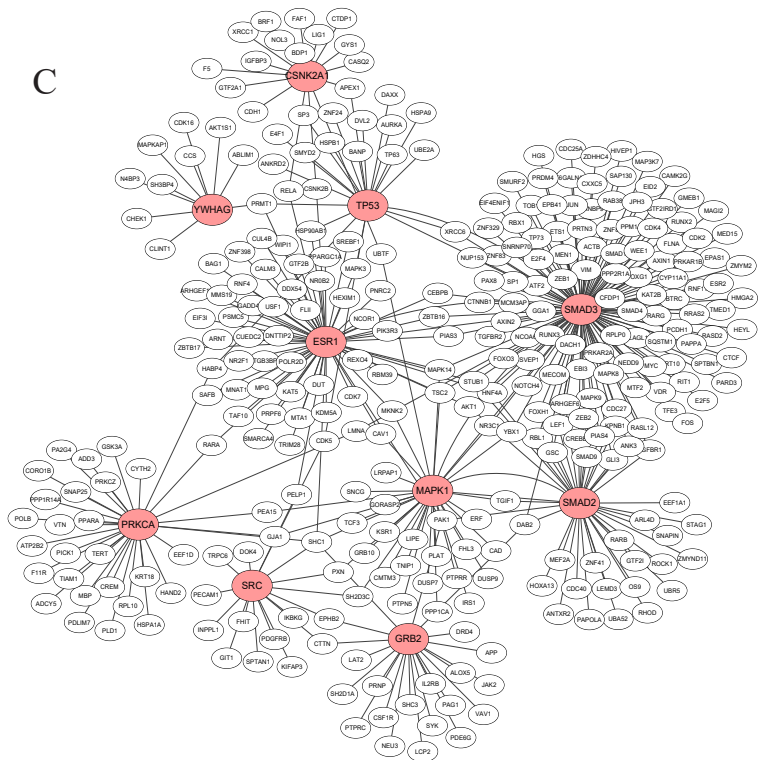

D

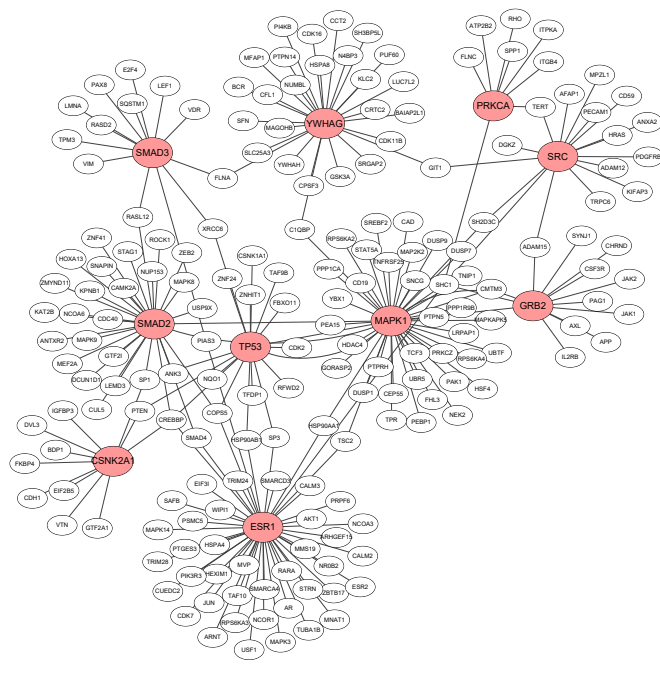

Supplement: Supplementary file 11 — Additional file 11. Figure S3. The network modules with the potential disease modules in LIHC Control network and Disease network. (A) The network modules among the top- 10 hub gene in Control network. (B) The network modules among the top- 10 hub gene in Disease network in sample LIHC_A9H1. (C) The network modules among the top- 10 hub gene in Disease network in sample LIHC _A69I. (D) The network modules among the top- 10 hub gene in Disease network in sample LIHC _AAC9. [file 12859_2022_4772_MOESM11_ESM.pdf]

A

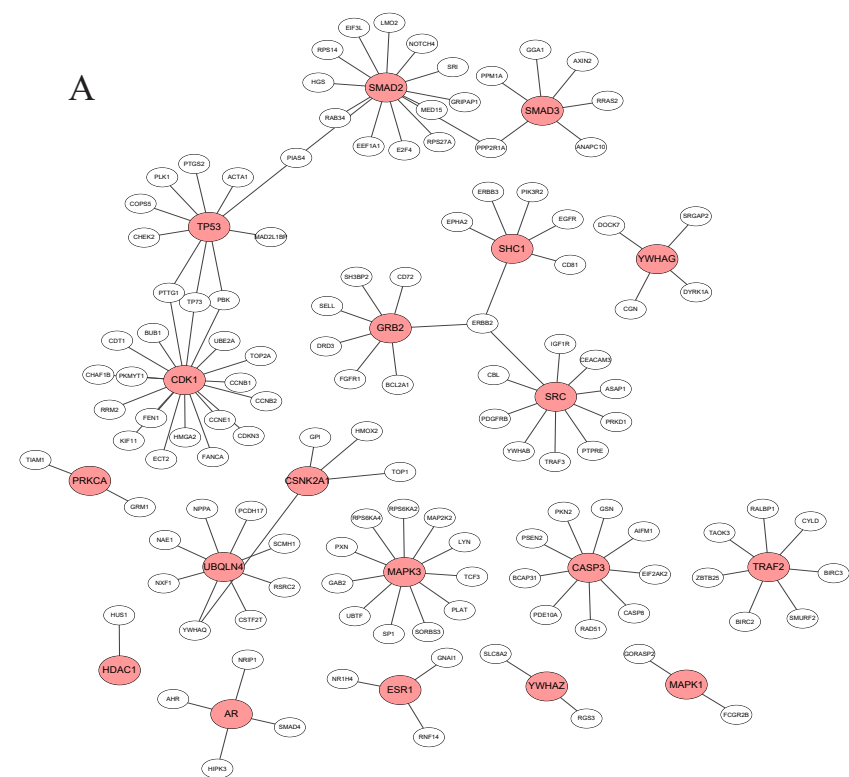

B

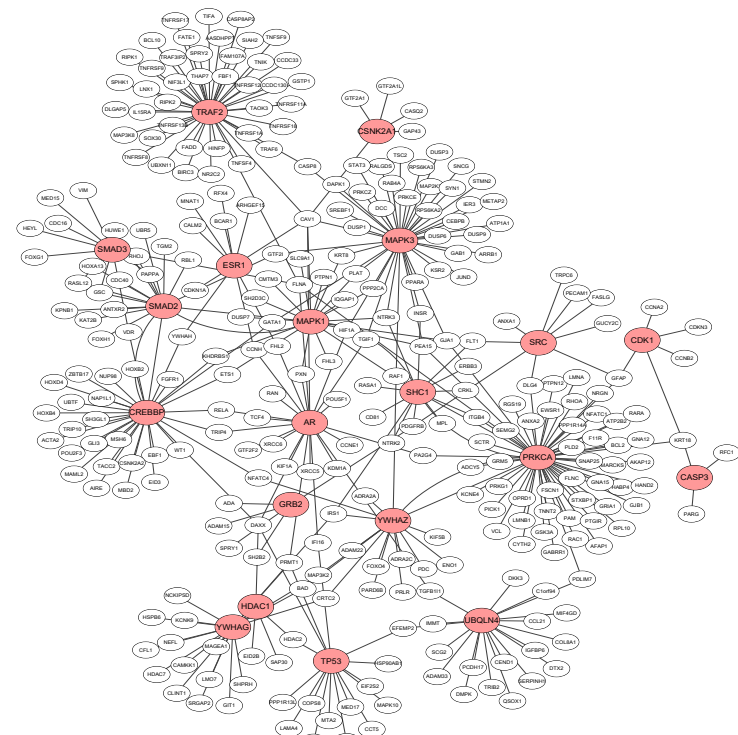

C

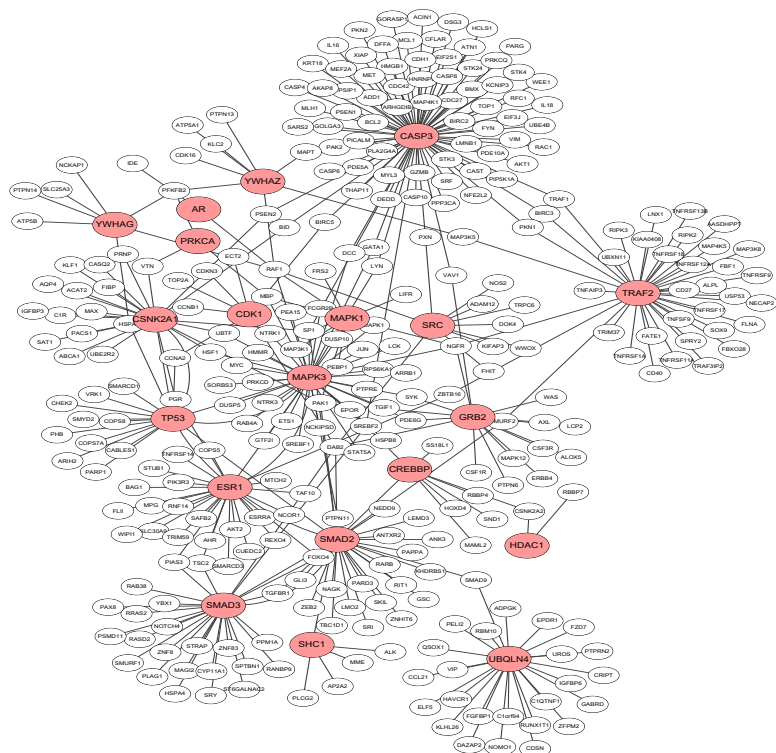

D

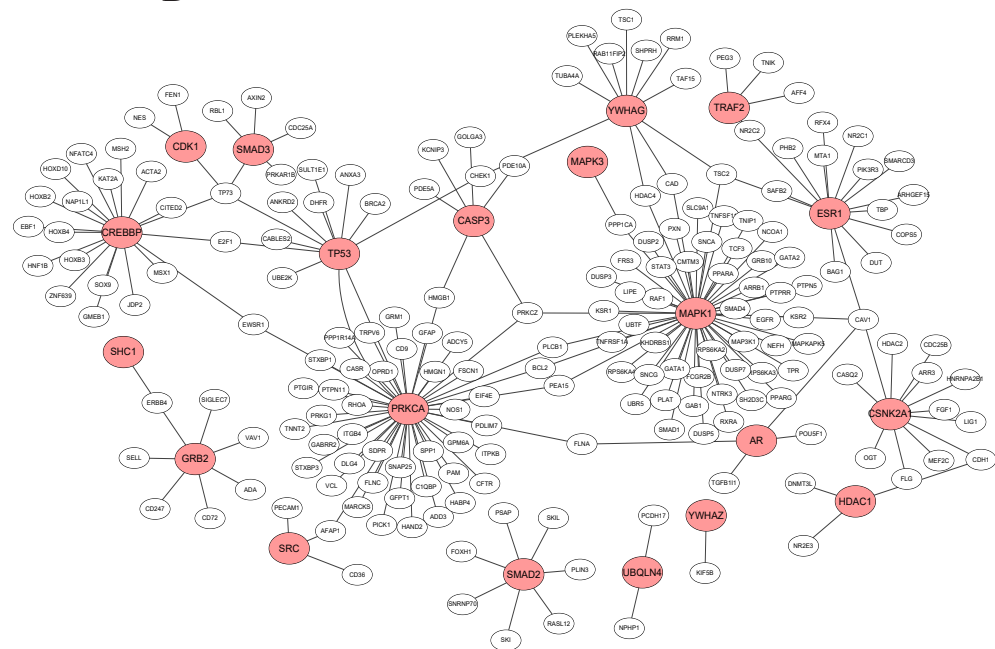

Supplement: Supplementary file 12 — Additional file 12. Figure S4. The network modules with the potential disease modules in LIHC Control network and Disease network. (A) The network modules among the top- 20 hub gene in Control network. (B) The network modules among the top- 20 hub gene in Disease network in sample LIHC_A110. (C) The network modules among the top- 20 hub gene in Disease network in sample LIHC _A520. (D) The network modules among the top- 20 hub gene in Disease network in sample LIHC _AA0V. [file 12859_2022_4772_MOESM12_ESM.pdf]

A

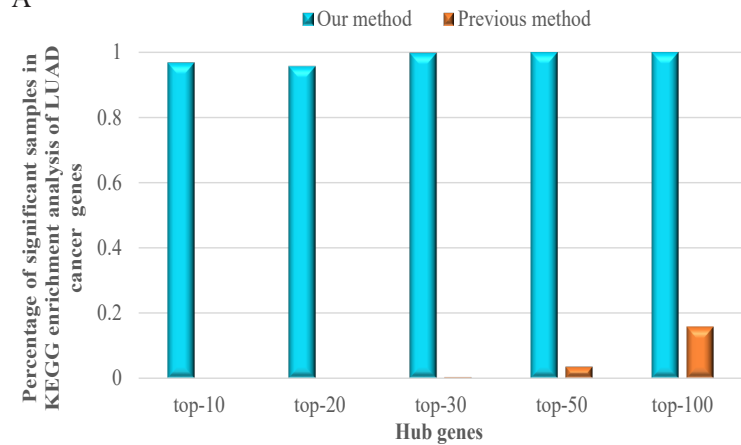

B

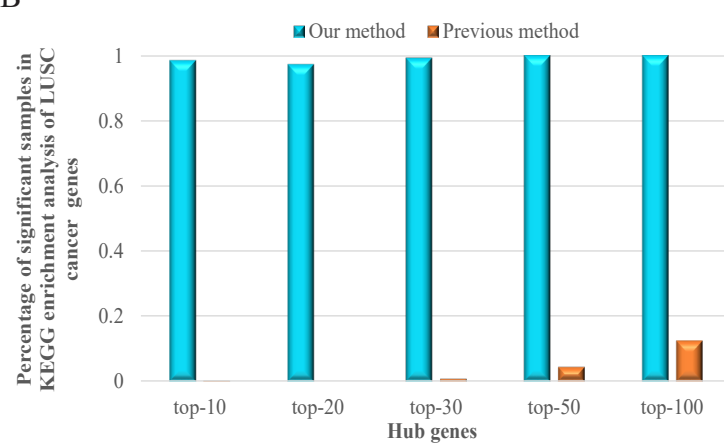

C

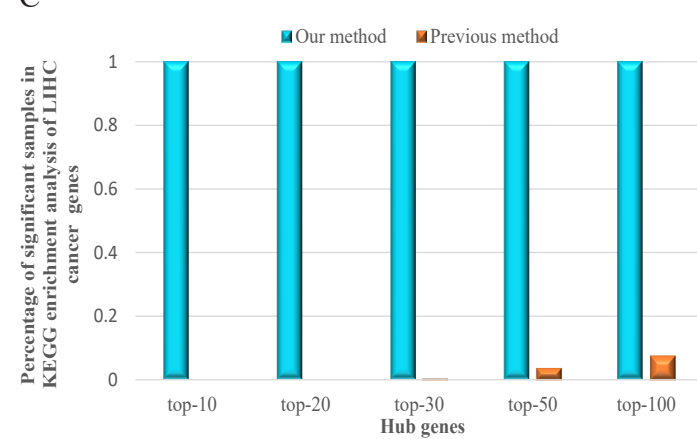

D

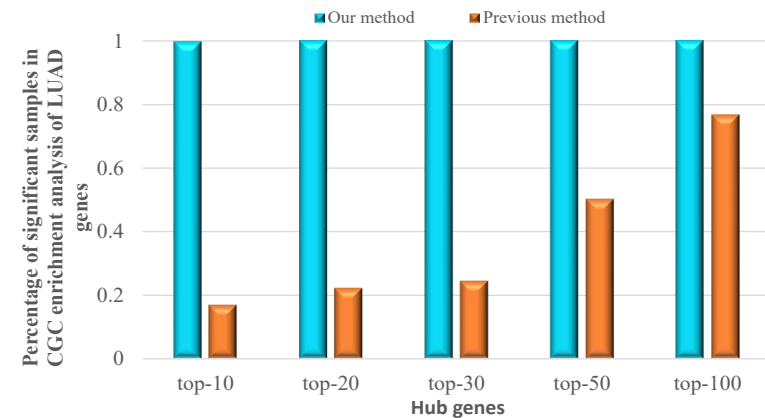

E

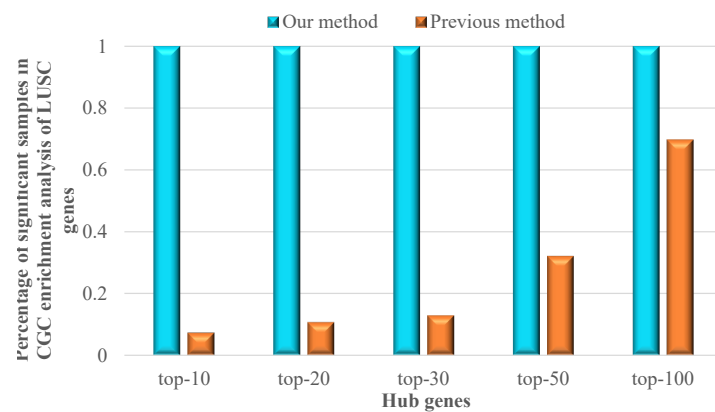

F

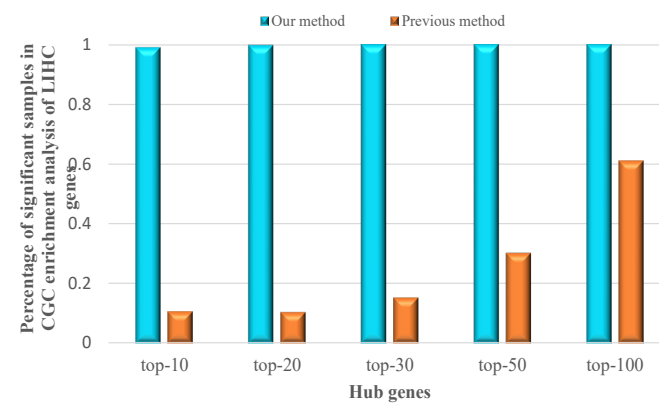

Supplement: Supplementary file 13 — Additional file 13. Figure S5. The enrichment in KEGG pathway and CGC database compared with our method and SSN method. (A)The proportion of significant samples in the enrichment analysis of top- 100, 50, 30, 20 and 10 highest degree genes for LUAD DSSN in the KEGG pathway and compare with the previous method. The x-axis is the hub genes and the y-axis is the percentage of significant samples in KEGG enrichment analysis. (B) The proportion of significant samples in the enrichment analysis of top- 100, 50, 30, 20 and 10 highest degree genes for LUSC DSSN in the KEGG pathway and compare with the previous method. The x-axis is the hub genes and the y-axis is the percentage of significant samples in KEGG enrichment analysis. (C) The proportion of significant samples in the enrichment analysis of top- 100, 50, 30, 20 and 10 highest degree genes for LIHC DSSN in the KEGG pathway and compare with the previous method. The x-axis is the hub genes and the y-axis is the percentage of significant samples in KEGG enrichment analysis. (D) The proportion of significant samples in the enrichment analysis of top- 100, 50, 30, 20 and 10 highest degree genes for LUAD DSSN in the CGC database and compare with the previous method. The x-axis is the hub genes of cancer and the y-axis is the percentage of significant samples in CGC enrichment analysis. (E) The proportion of significant samples in the enrichment analysis of top- 100, 50, 30, 20 and 10 highest degree genes for LUSC DSSN in the CGC database and compare with the previous method. The x-axis is the hub genes of cancer and the y-axis is the percentage of significant samples in CGC enrichment analysis. (F) The proportion of significant samples in the enrichment analysis of top- 100, 50, 30, 20 and 10 highest degree genes for LIHC DSSN in the CGC database and compare with the previous method. The x-axis is the hub genes of cancer and the y-axis is the percentage of significant samples in CGC enrichment analysis. [file 12859_2022_4772_MOESM13_ESM.pdf]
