# Supplementary material for: Identifying network biomarkers of cancer by sample-specific differential network
Source: BMC Bioinformatics. 2022 Jun 15;23:230. doi: 10.1186/s12859-022-04772-1 (PMC9202129; doi:10.1186/s12859-022-04772-1)
Supplement: Supplementary file 14 — Additional file 14. Figure S6. Survival curve for BRCA and LIHC. (A) Survival curve for BRCA survival analysis when using the most frequent 30 repetition hub genes to divide tumor samples into two groups. (B) Survival curve for BRCA survival analysis when using the most frequent 50 repetition hub genes to divide tumor samples into two groups. (C) Survival curve for LIHC survival analysis when using the most frequent 30 repetition hub genes to divide tumor samples into two groups. (D) Survival curve for LIHC survival analysis when using the most frequent 50 repetition hub genes to divide tumor samples into two groups. (E) Survival curve for LUAD survival analysis when using the most frequent 30 repetition hub genes to divide tumor samples into two groups. (F) Survival curve for LUSC survival analysis when using the most frequent 20 repetition hub genes to divide tumor samples into two groups. [file 12859_2022_4772_MOESM14_ESM.pdf]

A

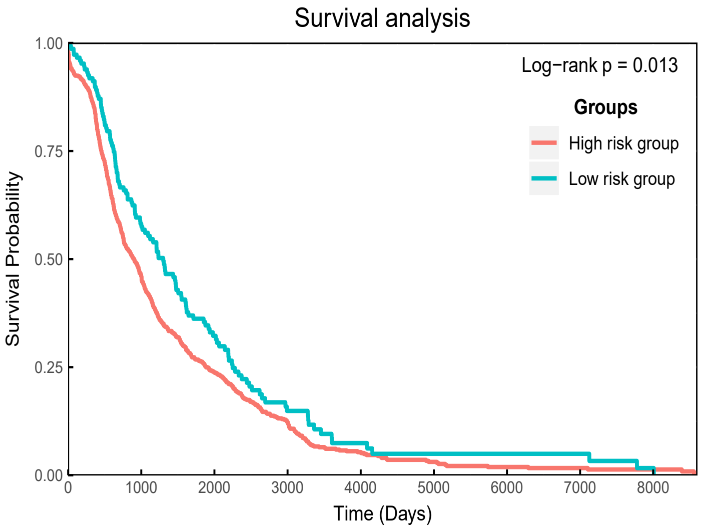

The Survival Curve Of BRCA

B

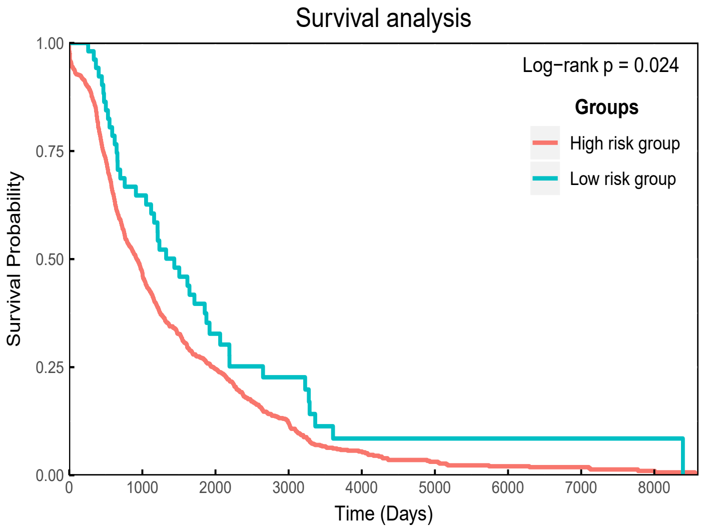

The Survival Curve Of BRCA

C

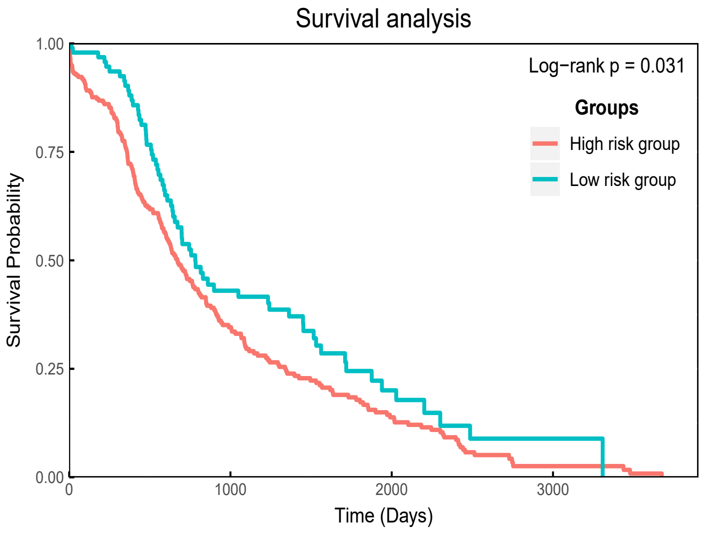

The Survival Curve Of LIHC

D

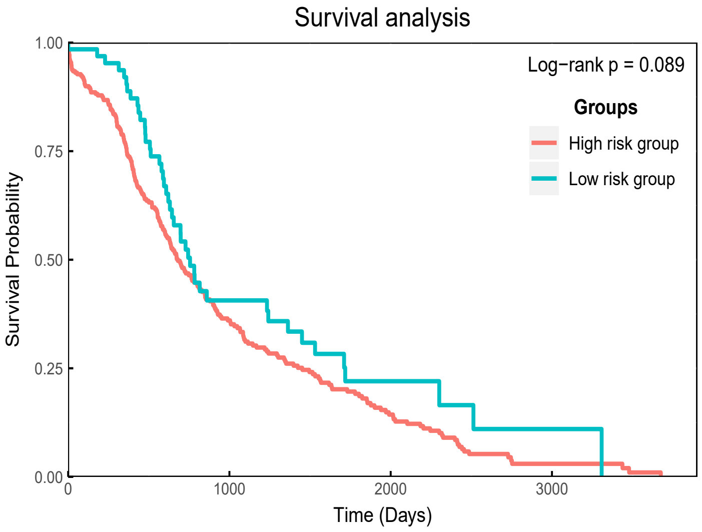

The Survival Curve Of LIHC

E

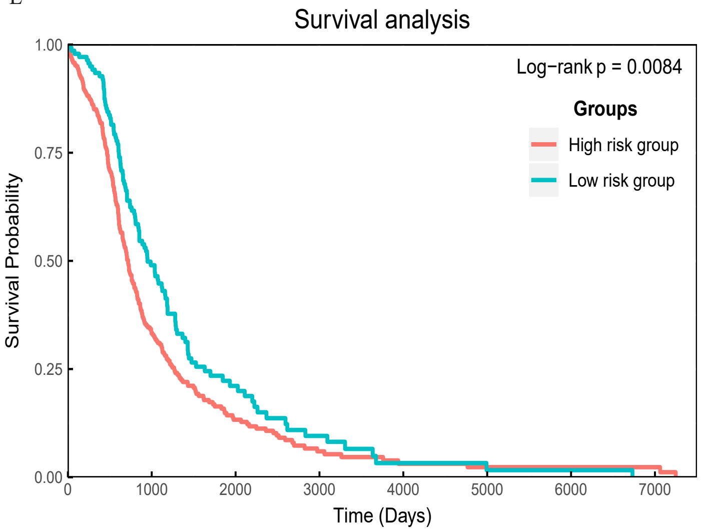

The Survival Curve Of LUAD

F

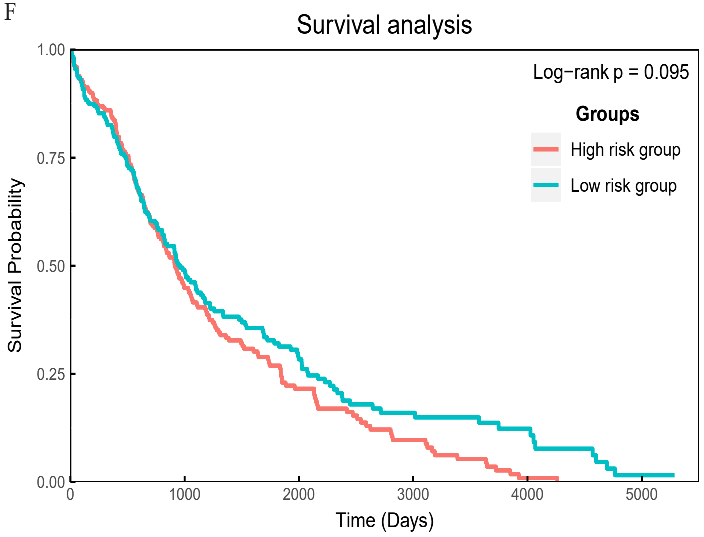

The Survival Curve Of LUSC
