# Supplementary material for: Identifying network biomarkers of cancer by sample-specific differential network
Source: BMC Bioinformatics. 2022 Jun 15;23:230. doi: 10.1186/s12859-022-04772-1 (PMC9202129; doi:10.1186/s12859-022-04772-1)
Supplement: Supplementary file 15 — Additional file 15. Summary documentation of Supplementary Information. [file 12859_2022_4772_MOESM15_ESM.docx]

**Note S1: Relationship between** $\boldsymbol{\Delta PCC}$

Assume that $X=\left[x_{1},\ldots,x_{n}\right]$ and $Y=\left[y_{1},\ldots,y_{n}\right]$ are two number vectors with length *n*, where $x_{i}$ is a real number and the *i*th $\left(1\leq i\leq n\right)$ element of *X*, $y_{j}$ is a real number and the *j*th $\left(1\leq j\leq n\right)$ element of *Y*. Here, *n* can be considered to be the number of the reference samples, and $x_{i}$, $y_{j}$ represent the expression levels of two molecules *X* and *Y* for sample *i* and *j*, respectively. Then, the *PCC* (Pearson correlation coefficient) for vectors *X* and *Y* can be defined as follows:

$\begin{matrix} R_{n}=\frac{\sum_{i=1}^{n} \left(x_{i}-\bar{x}\right)\left(y_{i}-\bar{y}\right)}{\sqrt{\sum_{i=1}^{n} \left(x_{i}-\bar{x}\right)^{2}\sum_{i=1}^{n} \left(y_{i}-\bar{y}\right)^{2}}} \\ =\frac{n\sum_{i=1}^{n} x_{i}y_{i}-\sum_{i=1}^{n} x_{i}\sum_{i=1}^{n} y_{i}}{\sqrt{n\sum_{i=1}^{n} x_{i}^{2}-\left(\sum_{i=1}^{n} x_{i}\right)^{2}}\sqrt{n\sum_{i=1}^{n} y_{i}^{2}-\left(\sum_{i=1}^{n} y_{i}\right)^{2}}} \\ =\frac{E\left(XY\right)-E\left(X\right)E\left(Y\right)}{\sigma_{X}\sigma_{Y}} \end{matrix}$,

where $R_{n}$ is the $PCC_{n}$ for *n* samples of the reference group, and

$\bar{x}=\frac{1}{n}\sum_{i=1}^{n} x_{i}$, $\bar{y}=\frac{1}{n}\sum_{i=1}^{n} y_{i}$.

We add two new samples $S_{a}$ with expression $\left(x_{a},y_{a}\right)$ and $S_{b}$ with expression $\left(x_{b},y_{b}\right)$ for gene *X* and *Y*. The two samples were added into reference samples to form new vector pairs $\left[\left(X,x_{a}\right),\left(Y,y_{a}\right)\right]$ and $\left[\left(X,x_{b}\right),\left(Y,y_{b}\right)\right]$. Then the *PCC*s between vectors $\left(X,x_{a}\right)$ and $\left(Y,y_{a}\right)$, and between vectors $\left(X,x_{b}\right)$ and $\left(Y,y_{b}\right)$ with the length (*n+1*) are calculated as and $R_{nb}$ as follow:

$R_{na}=\frac{\left(n+1\right)\sum_{i=1}^{n+1} x_{ia}y_{ia}-\sum_{i=1}^{n+1} x_{ia}\sum_{i=1}^{n+1} y_{ia}}{\left(n+1\right)^{2}\sigma_{X_{a}}\sigma_{Y_{a}}}$,

$R_{nb}=\frac{\left(n+1\right)\sum_{i=1}^{n\text{+}1} x_{ib}Y_{ib}-\sum_{i=1}^{n+1} x_{ib}\sum_{i=1}^{n+1} y_{ib}}{\left(n+1\right)^{2}\sigma_{X_{b}}\sigma_{Y_{b}}}$,

where $R_{na}$ and $R_{nb}$ are the $PCC_{n+1}$ for (*n+1*) samples, $\sum_{i=1}^{n+1} x_{ia}y_{ia}$ and $\sum_{i=1}^{n\text{+}1} x_{ib}y_{ib}$ are the sum of $x_{i}y_{i}$, after adding $\left(x_{a},y_{a}\right)$ and $\left(x_{b},y_{b}\right)$. $\sum_{i=1}^{n+1} x_{ia}$ and $\sum_{i=1}^{n+1} x_{ib}$ are the sum of $x_{i}$, after adding $x_{a}$ and $x_{b}$. $\sum_{i=1}^{n+1} y_{ia}$ and $\sum_{i=1}^{n+1} y_{ib}$ are the sum of $y_{i}$, after adding $y_{a}$ and $y_{b}$. $\sigma_{X_{a}}$ and $\sigma_{X_{b}}$ are the standard deviation of $\left\{x_{n}\right\}$, after adding $x_{a}$ and $x_{b}$. $\sigma_{Y_{a}}$ and $\sigma_{Y_{b}}$ are the standard deviation of $\left\{y_{n}\right\}$, after adding $y_{a}$ and $y_{b}$. The difference between the *PCCs* after adding new variables are $\Delta_{na}=R_{na}-R_{n}$ and $\Delta_{nb}=R_{nb}-R_{n}$ respectively.

Let

$\Delta_{na}\geq\Delta_{nb}$, S1

that is

$R_{na}-R_{n}\geq R_{nb}-R_{n}$,

$R_{na}-R_{nb}\geq0$,

we have

$\frac{\left(n+1\right)\sum_{i=1}^{n+1} x_{ia}y_{ia}-\sum_{i=1}^{n+1} x_{ia}\sum_{i=1}^{n+1} y_{ia}}{\left(n+1\right)^{2}\sigma_{X_{a}}\sigma_{Y_{a}}}-\frac{\left(n+1\right)\sum_{i=1}^{n\text{+}1} x_{ib}y_{ib}-\sum_{i=1}^{n+1} x_{ib}\sum_{i=1}^{n+1} y_{ib}}{\left(n+1\right)^{2}\sigma_{X_{b}}\sigma_{Y_{b}}}\geq0$,

that is

$\begin{matrix} \left(n+1\right)\left(\sum_{i=1}^{n+1} x_{ia}y_{ia}\sigma_{X_{b}}\sigma_{Y_{b}}-\sum_{i=1}^{n\text{+}1} x_{ib}y_{ib}\sigma_{X_{a}}\sigma_{Y_{a}}\right) \\ +\left(\sum_{i=1}^{n+1} x_{ib}\sum_{i=1}^{n+1} y_{ib}\sigma_{X_{a}}\sigma_{Y_{a}}-\sum_{i=1}^{n+1} x_{ia}\sum_{i=1}^{n+1} y_{ia}\sigma_{X_{b}}\sigma_{Y_{b}}\right)\geq0 \end{matrix}$ , S2

If

$\sum_{i=1}^{n+1} x_{ia}y_{ia}\sigma_{X_{b}}\sigma_{Y_{b}}-\sum_{i=1}^{n\text{+}1} x_{ib}y_{ib}\sigma_{X_{a}}\sigma_{Y_{a}}\geq0$,

and

$\sum_{i=1}^{n+1} x_{ib}\sum_{i=1}^{n+1} y_{ib}\sigma_{X_{a}}\sigma_{Y_{a}}-\sum_{i=1}^{n+1} x_{ia}\sum_{i=1}^{n+1} y_{ia}\sigma_{X_{b}}\sigma_{Y_{b}}\geq0$,

then the equation S2 is tenable.

That means the sufficient conditions of equation S1 are

$\frac{\sum_{i=1}^{n+1} x_{ia}y_{ia}}{\sum_{i=1}^{n\text{+}1} x_{ib}y_{ib}}\geq\frac{\sigma_{X_{a}}\sigma_{Y_{a}}}{\sigma_{X_{b}}\sigma_{Y_{b}}}$,

$\frac{\sum_{i=1}^{n+1} x_{ia}\sum_{i=1}^{n+1} y_{ia}}{\sum_{i=1}^{n+1} x_{ib}\sum_{i=1}^{n+1} y_{ib}}\leq\frac{\sigma_{X_{a}}\sigma_{Y_{a}}}{\sigma_{X_{b}}\sigma_{Y_{b}}}$.

Now we have another two vectors $X'=\left[x_{1}^{'},x_{2}^{'},\ldots,x_{m}^{'}\right]$ and $Y'=\left[y_{1}^{'},y_{2}^{'},\ldots,y_{m}^{'}\right]$ with length *m*, where $x_{i}^{'}$ is a real number and the *i*th $\left(1\leq i\leq m\right)$ element of $X'$, $y_{j}$ is a real number and the *j*th $\left(1\leq j\leq m\right)$ element of gene $Y'$. Here, *m* can be considered to be the number of the reference samples, $x_{i}^{'}$ and $y_{j}^{'}$ represent the expression levels of two molecules $X'$ and $Y'$, respectively. Then, the *PCC* for vectors $X'$ and $Y'$ can be defined as follows:

$\begin{matrix} R_{m}=\frac{\sum_{i=1}^{m} \left(x_{i}^{'}-\overline{x'}\right)\left(y_{i}-\overline{y'}\right)}{\sqrt{\sum_{i=1}^{m} \left(x_{i}^{'}-\overline{x'}\right)^{2}\sum_{i=1}^{m} \left(y_{i}-\overline{y'}\right)^{2}}} \\ =\frac{m\sum_{i=1}^{m} x_{i}^{'}y_{i}^{'}-\sum_{i=1}^{m} x_{i}^{'}\sum_{i=1}^{m} y_{i}^{'}}{\sqrt{m\sum_{i=1}^{m} x_{i}^{2}-\left(\sum_{i=1}^{m} x_{i}\right)^{2}}\sqrt{m\sum_{i=1}^{m} y_{i}^{2}-\left(\sum_{i=1}^{m} y_{i}\right)^{2}}} \\ =\frac{E\left(X'Y'\right)-E\left(X'\right)E\left(Y'\right)}{\sigma_{X'}\sigma_{Y'}} \end{matrix}$,

where $R_{m}$ is the $PCC_{m}$ for *m* samples of the reference group, and

$\overline{x'}=\frac{1}{m}\sum_{i=1}^{m} x_{i}^{'}$, $\overline{y'}=\frac{1}{m}\sum_{i=1}^{m} y_{i}^{'}$.

Then we add two new samples $S_{a}$ with expression $\left(x_{a},y_{a}\right)$ and $S_{b}$ with expression $\left(x_{b},y_{b}\right)$ for gene $X'$ and $Y'$. The two samples were added into reference samples to form new vector pairs $\left[\left(X',x_{a}\right),\left(Y',y_{a}\right)\right]$ and $\left[\left(X',x_{b}\right),\left(Y',y_{b}\right)\right]$. Then the *PCCs* between vectors $\left(X',x_{a}\right)$ and $\left(Y',y_{a}\right)$, and between vectors $\left(X',x_{b}\right)$ and $\left(Y',y_{b}\right)$ with the length (*m+1*) are calculated as $R_{ma}$ and $R_{mb}$ as follows:

$R_{ma}=\frac{\left(m+1\right)\sum_{i=1}^{m+1} x_{ia}^{'}y_{ia}^{'}-\sum_{i=1}^{m+1} x_{ia}^{'}\sum_{i=1}^{m+1} y_{ia}^{'}}{\left(m+1\right)^{2}\sigma_{X'_{a}}\sigma_{Y'_{a}}}$,

$R_{mb}=\frac{\left(m+1\right)\sum_{i=1}^{m+1} x_{ib}^{'}y_{ib}^{'}-\sum_{i=1}^{m+1} x_{ib}^{'}\sum_{i=1}^{m+1} y_{ib}^{'}}{\left(m+1\right)^{2}\sigma_{X'_{b}}\sigma_{Y'_{b}}}$,

where $R_{ma}$ and $R_{mb}$ are $PCC_{m+1}$ for $\left(m+1\right)$ samples, $\sum_{i=1}^{m+1} x_{ia}^{'}y_{ia}^{'}$ and $\sum_{i=1}^{m+1} x_{ib}^{'}y_{ib}^{'}$ are the sum of $x_{i}^{'}y_{i}^{'}$, after adding $\left(x_{a},y_{a}\right)$ and $\left(x_{b},y_{b}\right)$. $\sum_{i=1}^{m+1} x_{ia}^{'}$ and $\sum_{i=1}^{m+1} x_{ib}^{'}$ are the sum of $x_{i}^{'}$, after adding $x_{a}$ and $x_{b}$. $\sum_{i=1}^{m+1} y_{ia}^{'}$ and $\sum_{i=1}^{m+1} y_{ib}^{'}$ are the sum of $y_{i}^{'}$, after adding $y_{a}$ and $y_{b}$. $\sigma_{X'_{a}}$ and $\sigma_{Y'_{b}}$ are the standard deviation of $\left\{x_{m}^{'}\right\}$, after adding $x_{a}$ and $x_{b}$. $\sigma_{Y'_{a}}$ and $\sigma_{Y'_{b}}$ are the standard deviation of $\left\{y_{m}^{'}\right\}$, after adding $y_{a}$ and $y_{b}$. The difference between the *PCCs* after adding new variables are $\Delta_{ma}=R_{ma}-R_{m}$, $\Delta_{mb}=R_{mb}-R_{m}$, respectively.

If

$\frac{\sum_{i=1}^{m+1} x_{ia}^{'}y_{ia}^{'}}{\sum_{i=1}^{m+1} x_{ib}^{'}y_{ib}^{'}}\geq\frac{\sigma_{X'_{a}}\sigma_{Y'_{a}}}{\sigma_{X'_{b}}\sigma_{Y'_{b}}}$,

and

$\frac{\sum_{i=1}^{m+1} x_{ia}^{'}\sum_{i=1}^{m+1} y_{ia}^{'}}{\sum_{i=1}^{m+1} x_{ib}^{'}\sum_{i=1}^{m+1} y_{ib}^{'}}\leq\frac{\sigma_{X'_{a}}\sigma_{Y'_{a}}}{\sigma_{X'_{b}}\sigma_{Y'_{b}}}$,

then

$\sum_{i=1}^{m+1} x_{ia}^{'}y_{ia}^{'}\sigma_{X'_{b}}\sigma_{Y'_{b}}-\sum_{i=1}^{m+1} x_{ib}^{'}y_{ib}^{'}\sigma_{X'_{a}}\sigma_{Y'_{a}}\geq0$,

and

$\sum_{i=1}^{m+1} x_{ib}^{'}\sum_{i=1}^{m+1} y_{ib}^{'}\sigma_{X'_{a}}\sigma_{Y'_{a}}-\sum_{i=1}^{m+1} x_{ia}^{'}\sum_{i=1}^{m+1} y_{ia}^{'}\sigma_{X'_{b}}\sigma_{Y'_{b}}\geq0$,

that means

$\begin{matrix} \left(m+1\right)\left(\sum_{i=1}^{m+1} x_{ia}^{'}y_{ia}^{'}\sigma_{X'_{b}}\sigma_{Y'_{b}}-\sum_{i=1}^{m+1} x_{ib}^{'}y_{ib}^{'}\sigma_{X'_{a}}\sigma_{Y'_{a}}\right) \\ +\left(\sum_{i=1}^{m+1} x_{ib}^{'}\sum_{i=1}^{m+1} y_{ib}^{'}\sigma_{X'_{a}}\sigma_{Y'_{a}}-\sum_{i=1}^{m+1} x_{ia}^{'}\sum_{i=1}^{m+1} y_{ia}^{'}\sigma_{X'_{b}}\sigma_{Y'_{b}}\right)\geq0 \end{matrix}$,

$\frac{\left(m+1\right)\sum_{i=1}^{m+1} x_{ia}^{'}y_{ia}^{'}-\sum_{i=1}^{m+1} x_{ia}^{'}\sum_{i=1}^{m+1} y_{ia}^{'}}{\left(m+1\right)^{2}\sigma_{X'_{a}}\sigma_{Y'_{a}}}-\frac{\left(m+1\right)\sum_{i=1}^{m+1} x_{ib}^{'}\sum_{i=1}^{m+1} y_{ib}^{'}-\sum_{i=1}^{m+1} x_{ib}^{'}y_{ib}^{'}}{\left(m+1\right)^{2}\sigma_{X'_{b}}\sigma_{Y'_{b}}}\geq0$,

that is

$R_{ma}-R_{m}\geq R_{mb}-R_{m}$,

$\Delta_{ma}\geq\Delta_{mb}$.

Therefore, the sufficient conditions $\Delta_{ma}\geq\Delta_{mb}$ are

$\frac{\sum_{i=1}^{m+1} x_{ia}^{'}y_{ia}^{'}}{\sum_{i=1}^{m+1} x_{ib}^{'}y_{ib}^{'}}\geq\frac{\sigma_{X'_{a}}\sigma_{Y'_{a}}}{\sigma_{X'_{b}}\sigma_{Y'_{b}}}$,

$\frac{\sum_{i=1}^{m+1} x_{ia}^{'}\sum_{i=1}^{m+1} y_{ia}^{'}}{\sum_{i=1}^{m+1} x_{ib}^{'}\sum_{i=1}^{m+1} y_{ib}^{'}}\leq\frac{\sigma_{X'_{a}}\sigma_{Y'_{a}}}{\sigma_{X'_{b}}\sigma_{Y'_{b}}}$.

Conclusion 1: If $n,m\to\infty$, and $\Delta_{na}\geq\Delta_{nb}$, then $\Delta_{ma}\geq\Delta_{mb}$.

Proof:

We have $\Delta_{na}\geq\Delta_{nb}$, that is

$\frac{\sum_{i=1}^{n+1} x_{ia}y_{ia}}{\sum_{i=1}^{n\text{+}1} x_{ib}y_{ib}}\geq\frac{\sigma_{X_{a}}\sigma_{Y_{a}}}{\sigma_{X_{b}}\sigma_{Y_{b}}}$,

$\frac{\sum_{i=1}^{n+1} x_{ia}\sum_{i=1}^{n+1} y_{ia}}{\sum_{i=1}^{n+1} x_{ib}\sum_{i=1}^{n+1} y_{ib}}\leq\frac{\sigma_{X_{a}}\sigma_{Y_{a}}}{\sigma_{X_{b}}\sigma_{Y_{b}}}$,

while the sufficient and unnecessary conditions of $\Delta_{ma}\geq\Delta_{mb}$ is

$\frac{\sum_{i=1}^{m+1} x_{ia}^{'}y_{ia}^{'}}{\sum_{i=1}^{m+1} x_{ib}^{'}y_{ib}^{'}}\geq\frac{\sigma_{X'_{a}}\sigma_{Y'_{a}}}{\sigma_{X'_{b}}\sigma_{Y'_{b}}}$, S3

$\frac{\sum_{i=1}^{m+1} x_{ia}^{'}\sum_{i=1}^{m+1} y_{ia}^{'}}{\sum_{i=1}^{m+1} x_{ib}^{'}\sum_{i=1}^{m+1} y_{ib}^{'}}\leq\frac{\sigma_{X'_{a}}\sigma_{Y'_{a}}}{\sigma_{X'_{b}}\sigma_{Y'_{b}}}$. S4

When $n\to\infty$,

$\mathop{lim}\limits_{n\to\infty} \frac{\sum_{i=1}^{n+1} x_{ia}y_{ia}}{\sum_{i=1}^{n\text{+}1} x_{ib}y_{ib}}\text{=}\mathop{lim}\limits_{n\to\infty} \frac{n\bar{x}\bar{y}+x_{a}x_{a}}{n\bar{x}\bar{y}+x_{b}x_{b}}=\mathop{lim}\limits_{n\to\infty} \frac{\bar{x}\bar{y}+\frac{x_{a}x_{a}}{n}}{\bar{x}\bar{y}+\frac{x_{b}x_{b}}{n}}=1$,

$\mathop{lim}\limits_{n\to\infty} \frac{\sum_{i=1}^{n+1} x_{ia}\sum_{i=1}^{n+1} y_{ia}}{\sum_{i=1}^{n+1} x_{ib}\sum_{i=1}^{n+1} y_{ib}}\text{=}\mathop{lim}\limits_{n\to\infty} \frac{\left(n\bar{x}+x_{a}\right)\left(n\bar{y}+y_{a}\right)}{\left(n\bar{x}+x_{b}\right)\left(n\bar{y}+y_{b}\right)}=\mathop{lim}\limits_{n\to\infty} \frac{\left(\bar{x}+\frac{x_{a}}{n}\right)\left(\bar{y}+\frac{y_{a}}{n}\right)}{\left(\bar{x}+\frac{x_{b}}{n}\right)\left(\bar{y}+\frac{y_{b}}{n}\right)}=1$,

$\begin{matrix} \mathop{lim}\limits_{n\to\infty} \frac{\sigma_{X_{a}}\sigma_{Y_{a}}}{\sigma_{X_{b}}\sigma_{Y_{b}}}=\mathop{lim}\limits_{n\to\infty} \frac{\sqrt{\left[\left(n+1\right)\left(n\overline{x^{2}}+x_{a}^{2}\right)-\left(n\bar{x}+x_{a}\right)^{2}\right]\left[\left(n+1\right)\left(n\overline{y^{2}}+y_{a}^{2}\right)-\left(n\bar{y}+y_{a}\right)^{2}\right]}}{\sqrt{\left[\left(n+1\right)\left(n\overline{x^{2}}+x_{b}^{2}\right)-\left(n\bar{x}+x_{b}\right)^{2}\right]\left[\left(n+1\right)\left(n\overline{y^{2}}+y_{b}^{2}\right)-\left(n\bar{y}+y_{b}\right)^{2}\right]}} \\ =\mathop{lim}\limits_{n\to\infty} \frac{\sqrt{\left[\left(1+\frac{1}{n}\right)\left(\overline{x^{2}}+\frac{x_{a}^{2}}{n}\right)-\left(\bar{x}+\frac{x_{a}}{n}\right)^{2}\right]\left[\left(1+\frac{1}{n}\right)\left(\overline{y^{2}}+\frac{y_{a}^{2}}{n}\right)-\left(\bar{y}+\frac{y_{a}}{n}\right)^{2}\right]}}{\sqrt{\left[\left(1+\frac{1}{n}\right)\left(\overline{x^{2}}+\frac{x_{b}^{2}}{n}\right)-\left(\bar{x}+\frac{x_{b}}{n}\right)^{2}\right]\left[\left(1+\frac{1}{n}\right)\left(\overline{y^{2}}+\frac{y_{b}^{2}}{n}\right)-\left(\bar{y}+\frac{y_{b}}{n}\right)^{2}\right]}} \\ =1 \end{matrix}$,

where $n\bar{x}\bar{y}\text{=}\sum_{i=1}^{n} x_{i}y_{i}$, $\overline{x^{2}}=\frac{1}{n}\sum_{i=1}^{n} x_{i}^{2}$, $\overline{y^{2}}=\frac{1}{n}\sum_{i=1}^{n} y_{i}^{2}$.

When $m\to\infty$,

,

$\mathop{lim}\limits_{m\to\infty} \frac{\sum_{i=1}^{m+1} x_{ia}^{'}\sum_{i=1}^{m+1} y_{ia}^{'}}{\sum_{i=1}^{m+1} x_{ib}^{'}\sum_{i=1}^{m+1} y_{ib}^{'}}=\mathop{lim}\limits_{m\to\infty} \frac{\left(m\overline{x'}+x_{a}\right)\left(m\overline{y'}+y_{a}\right)}{\left(m\overline{x'}+x_{b}\right)\left(m\overline{y'}+y_{b}\right)}=\mathop{lim}\limits_{m\to\infty} \frac{\left(\overline{x'}+\frac{x_{a}}{m}\right)\left(\overline{y'}+\frac{y_{a}}{m}\right)}{\left(\overline{x'}+\frac{x_{b}}{m}\right)\left(\overline{y'}+\frac{y_{b}}{m}\right)}=1$,

$\begin{matrix} \mathop{lim}\limits_{m\to\infty} \frac{\sigma_{X'_{a}}\sigma_{Y'_{a}}}{\sigma_{X'_{b}}\sigma_{Y'_{b}}}\text{=}\mathop{lim}\limits_{m\to\infty} \frac{\sqrt{\left[\left(m+1\right)\left(m\overline{x'^{2}}+mx_{a}^{2}\right)-\left(m\overline{x'}+mx_{a}\right)^{2}\right]\left[\left(m+1\right)\left(m\overline{y'^{2}}+my_{a}^{2}\right)-\left(m\overline{y'}+my_{a}\right)^{2}\right]}}{\sqrt{\left[\left(m+1\right)\left(m\overline{x'^{2}}+mx_{b}^{2}\right)-\left(m\overline{x'}+mx_{b}\right)^{2}\right]\left[\left(m+1\right)\left(m\overline{y'^{2}}+my_{b}^{2}\right)-\left(m\overline{y'}+my_{b}\right)^{2}\right]}} \\ =\mathop{lim}\limits_{m\to\infty} \frac{\sqrt{\left[\left(1+\frac{1}{m}\right)\left(\overline{x'^{2}}+\frac{x_{a}^{2}}{m}\right)-\left(\overline{x'}+\frac{x_{a}}{m}\right)^{2}\right]\left[\left(1+\frac{1}{m}\right)\left(\overline{y'^{2}}+\frac{y_{a}^{2}}{m}\right)-\left(\overline{y'}+\frac{y_{a}}{m}\right)^{2}\right]}}{\sqrt{\left[\left(1+\frac{1}{m}\right)\left(\overline{x'^{2}}+\frac{x_{b}^{2}}{m}\right)-\left(\overline{x'}+\frac{x_{b}}{m}\right)^{2}\right]\left[\left(1+\frac{1}{m}\right)\left(\overline{y'^{2}}+\frac{y_{b}^{2}}{m}\right)-\left(\overline{y'}+\frac{y_{b}}{m}\right)^{2}\right]}} \\ =1 \end{matrix}$,

where $m\overline{x'y'}\text{=}\sum_{i=1}^{m} x_{i}^{'}y_{i}^{'}$, , .

That is

$\mathop{lim}\limits_{n\to\infty} \frac{\sum_{i=1}^{n+1} x_{ia}y_{ia}}{\sum_{i=1}^{n\text{+}1} x_{ib}y_{ib}}=\mathop{lim}\limits_{m\to\infty} \frac{\sum_{i=1}^{m+1} x_{ia}^{'}y_{ia}^{'}}{\sum_{i=1}^{m+1} x_{ib}^{'}y_{ib}^{'}}=1$,

$\mathop{lim}\limits_{n\to\infty} \frac{\sum_{i=1}^{n+1} x_{ia}\sum_{i=1}^{n+1} y_{ia}}{\sum_{i=1}^{n+1} x_{ib}\sum_{i=1}^{n+1} y_{ib}}\text{=}\mathop{lim}\limits_{m\to\infty} \frac{\sum_{i=1}^{m+1} x_{ia}^{'}\sum_{i=1}^{m+1} y_{ia}^{'}}{\sum_{i=1}^{m+1} x_{ib}^{'}\sum_{i=1}^{m+1} y_{ib}^{'}}=1$,

$\mathop{lim}\limits_{n\to\infty} \frac{\sigma_{X_{a}}\sigma_{Y_{a}}}{\sigma_{X_{b}}\sigma_{Y_{b}}}=\mathop{lim}\limits_{m\to\infty} \frac{\sigma_{X'_{a}}\sigma_{Y'_{a}}}{\sigma_{X'_{b}}\sigma_{Y'_{b}}}=1$,

So,

$\mathop{lim}\limits_{m\to\infty} \frac{\sum_{i=1}^{m+1} x_{ia}^{'}y_{ia}^{'}}{\sum_{i=1}^{m+1} x_{ib}^{'}y_{ib}^{'}}=\mathop{lim}\limits_{m\to\infty} \frac{\sigma_{X'_{a}}\sigma_{Y'_{a}}}{\sigma_{X'_{b}}\sigma_{Y'_{b}}}=1$,

$\mathop{lim}\limits_{m\to\infty} \frac{\sum_{i=1}^{m+1} x_{ia}^{'}\sum_{i=1}^{m+1} y_{ia}^{'}}{\sum_{i=1}^{m+1} x_{ib}^{'}\sum_{i=1}^{m+1} y_{ib}^{'}}=\mathop{lim}\limits_{m\to\infty} \frac{\sigma_{X'_{a}}\sigma_{Y'_{a}}}{\sigma_{X'_{b}}\sigma_{Y'_{b}}}=1$,

Therefore, equation S3 and S4 are tenable, which means the sufficient conditions of $\Delta_{m_{a}}\geq\Delta_{m_{b}}$are satisfied.

As a result, when $n,m\to\infty$, and $\Delta_{na}\geq\Delta_{nb}$, we have $\Delta_{ma}\geq\Delta_{mb}$.

Conclusion 2：If vector $X$ and vector $X'$ belong to one independent identically distributed random variables $\left\{S_{n}\right\}$, vector $Y$ and vector $Y'$ belong to another independent identically distributed random variables $\left\{W_{n}\right\}$, and $E\left(S_{n}^{4}\right)<\infty$, $E\left(W_{n}^{4}\right)<\infty$, $\Delta_{na}\geq\Delta_{nb}$, then we have $\Delta_{ma}\geq\Delta_{mb}$.

Proof:

We have $\Delta_{na}\geq\Delta_{nb}$, and its sufficient conditions are

$\frac{\sum_{i=1}^{n+1} x_{ia}y_{ia}}{\sum_{i=1}^{n\text{+}1} x_{ib}y_{ib}}\geq\frac{\sigma_{X_{a}}\sigma_{Y_{a}}}{\sigma_{X_{b}}\sigma_{Y_{b}}}$,

$\frac{\sum_{i=1}^{n+1} x_{ia}\sum_{i=1}^{n+1} y_{ia}}{\sum_{i=1}^{n+1} x_{ib}\sum_{i=1}^{n+1} y_{ib}}\leq\frac{\sigma_{X_{a}}\sigma_{Y_{a}}}{\sigma_{X_{b}}\sigma_{Y_{b}}}$.

Let

$Var\left(S_{n}\right)=\sigma_{1}^{2}$,

$Var\left(W_{n}\right)=\sigma_{2}^{2}$,

$E\left(S_{n}\right)=\mu_{1}$,

$E\left(W_{n}\right)=\mu_{2}$.

Note that

$\left(S_{n}-\mu_{1}\right)^{2}\text{，}n=1,2,...$,

then

$Var\left(S_{n}-\mu_{1}\right)^{2}=E\left(S_{n}-\mu_{1}\right)^{4}-\sigma_{1}^{4}$.

We have

$E\left(S_{n}^{4}\right)<\infty$,

so

$Var\left(S_{n}-\mu_{1}\right)^{2}<\infty$.

According to Chebyshev's Theorem, for any $\varepsilon>0$, we have

$\mathop{lim}\limits_{n\to\infty} P\left(\left|\frac{1}{n}\sum_{i=1}^{n} \left(S_{i}-\mu_{1}\right)^{2}-\frac{1}{n}\sum_{i=1}^{n} E\left(S_{i}-\mu_{1}\right)^{2}\right|\geq\varepsilon\right)=0$,

where $\frac{1}{n}\sum_{i=1}^{n} E\left(S_{i}-\mu_{1}\right)^{2}=\sigma_{1}^{2}$.

After adding $\left(x_{a},y_{a}\right)$, we have

$\mathop{lim}\limits_{n\to\infty} P\left(\left|\frac{1}{n+1}\sum_{i=1}^{n+1} \left(S_{i}-\mu_{1}\right)^{2}-\frac{1}{n+1}\sum_{i=1}^{n+1} E\left(S_{i}-\mu_{1}\right)^{2}\right|\geq\varepsilon\right)=0$.

Hence

$\mathop{lim}\limits_{n\to\infty} P\left(\left|\sigma_{X_{a}}^{2}-\sigma_{1}^{2}\right|\geq\varepsilon\right)=0$.

In the same way, after adding $\left(x_{b},y_{b}\right)$, we also have

$\mathop{lim}\limits_{n\to\infty} P\left(\left|\sigma_{X_{b}}^{2}-\sigma_{1}^{2}\right|\geq\varepsilon\right)=0$.

Similarly, it can be proved that $\left\{\left(W_{n}-\mu_{2}\right)^{2}\right\}$ also obeys Chebyshev's Theorem.

In the same way, we can get

$\mathop{lim}\limits_{m\to\infty} P\left(\left|\sigma_{X'_{a}}^{2}-\sigma_{1}^{2}\right|\geq\varepsilon\right)=0$,

$\mathop{lim}\limits_{m\to\infty} P\left(\left|\sigma_{X'_{b}}^{2}-\sigma_{1}^{2}\right|\geq\varepsilon\right)=0$,

$\mathop{lim}\limits_{n\to\infty} P\left(\left|\sigma_{Y_{a}}-\sigma_{2}^{2}\right|\geq\varepsilon\right)=0$,

$\mathop{lim}\limits_{n\to\infty} P\left(\left|\sigma_{Y_{b}}-\sigma_{2}^{2}\right|\geq\varepsilon\right)=0$,

$\mathop{lim}\limits_{m\to\infty} P\left(\left|\sigma_{Y'_{a}}-\sigma_{2}^{2}\right|\geq\varepsilon\right)=0$,

$\mathop{lim}\limits_{m\to\infty} P\left(\left|\sigma_{Y'_{b}}-\sigma_{2}^{2}\right|\geq\varepsilon\right)=0$.

Hence

$\frac{\sigma_{X_{a}}\sigma_{Y_{a}}}{\sigma_{X_{b}}\sigma_{Y_{b}}}=\frac{\sigma_{X'_{a}}\sigma_{Y'_{a}}}{\sigma_{X'_{b}}\sigma_{Y'_{b}}}$.

For another sequence $\left\{S_{n}\right\}$, according to Bernoulli's theorem,

$\mathop{lim}\limits_{n\to\infty} P\left(\left|\frac{1}{n}\sum_{i=1}^{n} x_{i}-\frac{1}{n}\sum_{i=1}^{n} E\left(x_{i}\right)\right|\geq\varepsilon\right)=0$,

where $\frac{1}{n}\sum_{i=1}^{n} E\left(x_{i}\right)\text{=}\mu_{1}$.

After adding $\left(x_{a},y_{a}\right)$, we have

$\mathop{lim}\limits_{n\to\infty} P\left(\left|\frac{1}{n+1}\sum_{i=1}^{n+1} x_{ia}-\frac{1}{n+1}\sum_{i=1}^{n+1} E\left(x_{i}\right)\right|\geq\varepsilon\right)=0$,

that is

$\mathop{lim}\limits_{n\to\infty} P\left(\left|\frac{1}{n+1}\sum_{i=1}^{n+1} x_{ia}-\mu_{1}\right|\geq\varepsilon\right)=0$.

In the same way, after adding $\left(x_{b},y_{b}\right)$, we also have

$\mathop{lim}\limits_{n\to\infty} P\left(\left|\frac{1}{n+1}\sum_{i=1}^{n\text{+}1} x_{ib}-\mu_{1}\right|\geq\varepsilon\right)=0$.

Similarly, it can be proved that $\left\{W_{n}\right\}$ also obeys Bernoulli's theorem

Therefore, in the same way, we can get

$\mathop{lim}\limits_{m\to\infty} P\left(\left|\frac{1}{m+1}\sum_{i=1}^{m\text{+}1} x_{ia}^{'}-\mu_{1}\right|\geq\varepsilon\right)=0$,

$\mathop{lim}\limits_{m\to\infty} P\left(\left|\frac{1}{m+1}\sum_{i=1}^{m\text{+}1} x_{ib}^{'}-\mu_{1}\right|\geq\varepsilon\right)=0$,

$\mathop{lim}\limits_{n\to\infty} P\left(\left|\frac{1}{n+1}\sum_{i=1}^{n\text{+}1} y_{ia}-\mu_{2}\right|\geq\varepsilon\right)=0$,

$\mathop{lim}\limits_{n\to\infty} P\left(\left|\frac{1}{n+1}\sum_{i=1}^{n\text{+}1} y_{ib}-\mu_{2}\right|\geq\varepsilon\right)=0$,

$\mathop{lim}\limits_{m\to\infty} P\left(\left|\frac{1}{m+1}\sum_{i=1}^{m\text{+}1} y_{ia}^{'}-\mu_{2}\right|\geq\varepsilon\right)=0$,

$\mathop{lim}\limits_{m\to\infty} P\left(\left|\frac{1}{m+1}\sum_{i=1}^{m\text{+}1} y_{ib}^{'}-\mu_{2}\right|\geq\varepsilon\right)=0$.

As a result

$\frac{\sum_{i=1}^{m+1} x_{ia}^{'}y_{ia}^{'}}{\sum_{i=1}^{m+1} x_{ib}^{'}y_{ib}^{'}}\geq\frac{\sigma_{X'_{a}}\sigma_{Y'_{a}}}{\sigma_{X'_{b}}\sigma_{Y'_{b}}}=\frac{\sigma_{X_{a}}\sigma_{Y_{a}}}{\sigma_{X_{b}}\sigma_{Y_{b}}}$,

$\frac{\sum_{i=1}^{m+1} x_{ia}^{'}\sum_{i=1}^{m+1} y_{ia}^{'}}{\sum_{i=1}^{m+1} x_{ib}^{'}\sum_{i=1}^{m+1} y_{ib}^{'}}\leq\frac{\sigma_{X'_{a}}\sigma_{Y'_{a}}}{\sigma_{X'_{b}}\sigma_{Y'_{b}}}=\frac{\sigma_{X_{a}}\sigma_{Y_{a}}}{\sigma_{X_{b}}\sigma_{Y_{b}}}$.

The sufficient conditions of $\Delta_{ma}\geq\Delta_{mb}$ are satisfied.

**Note S2: The roles of the background network**

In our study, we use the 24,991 genes in human. If each gene pair has to be calculated the Pearson correlation coefficient, which is a fully connected, then for one sample, the total of calculations is 24,991$\times$24,991=624,5500,081. It just the amount of calculation for one sample, will need to be calculated hundreds of millions of times. If thousands of samples have calculated like this way, the amount of calculation will be very huge. We tried to use the server to calculate, but failed to find that the efficiency is still not very high.

Besides, if consider fully connected and do not use the background network, the number of false positive gene pairs will also increase. For example, there are 4 genes, gene a, b, c and d, the positive gene pairs are gene a and gene b, gene b and gene c, gene c and gene d. However, if we use the fully connected, there is a linear relationship between gene pairs, then it could conclude that the gene a and gene c, gene a and gene d are also related. These are the false positive gene pairs. With the increase in the number of genes, the amount of false positive gene pairs are also increasing, which could eventually lead to false positive gene pairs accounting for half of the effective gene pairs.

Due to the above two unavoidably situations, we adopted the background network. Most of the gene pairs in the background network are verified by scientific and real experiments, and these gene pairs are truly related. Calculate the *PCC* of the gene pairs in the background network, on the one hand, can reduce the computational pressure. On the other hand, it can reduce the proportion of false positive gene pairs. Although we may lose some gene pairs, these gene pairs have truly correlation, because these are not in the background network, and are directly screened out. Compared with the huge false positives gene pairs, this loss is negligible. Certainly we can use the STRING database (<https://string-db.org/>) as the background network, it also a widely used, well-known and authoritative database. The results of using STRING and HPRD database have slightly differences, while is still similar. For this reason, using different background networks has slightly effect on the performance, and we chose the HPRD database.

**Note S3: Hub genes based on different reference networks**

The normal samples of GSE27342 and GSE63089 as two reference networks. For example, we add GSE27342 normal samples into reference networks and obtain the *s-PCC* between each pair of genes. If the relationship between a pair of genes is significant, this pair of genes can be linked as an edge in Control network. In the same way, we add GSE27342 disease samples in reference networks and contain Disease network. Hence, for one person, there is a Disease network and a Control network. For Disease network and Control network, there are six ways to obtain SSDN. The first kind of network is constructing based on specific genes in Disease and Control networks, the rest are constructing based on common genes in Disease and Control network, specific genes in Control networks, specific genes in Disease networks, genes only in Control networks, genes only in Disease networks. We selected the most frequent 300, 100, 50, 30, 20 and 10 repetition hub genes, compared whether these hub genes are common based on two reference networks, and get the Table S1 according to the results.

**Note S4: Compare DSSN and SSN method in enrichment analysis**

We compare the proportion of significant samples in the top- 100, 50, 30, 20 and 10 hub genes in KEGG pathway enrichment by DSSN. Comparing our method and SSN method^1^, which demonstrates that the DSSN is effective and useful in finding cancer pathway (Figure S1A-C).

At the same time, we also compare the proportion of significant samples in the top- 100, 50, 30, 20 and 10 degree genes in CGC enrichment by DSSN, which also implies that the DSSN is more powerful in disease gene enrichment (Figure S1D-F).

**Note S5: Compare DSSN and a network-based approach in enrichment analysis**

The previous work of Liu, X et al^2^, was to build a network using multiple normal samples of the same disease, and build a disease network using multiple disease samples. This is a network of the general commonality of the disease, which can reflect the general information of the disease, but cannot reflect the specific information of the individual. The SSDN method (our method) is a single-sample individual specific network. We take the normal state and disease state of the same cancer as a whole, and no longer calculate the single-sample network. Then we calculate the CGC enrichment and KEGG enrichment of the whole cancer. Although the SSDN method is used for single sample difference analysis, it turns out when treat the cancer as a whole, the enrichment analysis results are still good (Table S2, Table S3).

**Note S6: The classification for SSN method**

For BRCA, We use SSDN method, and select the gene pairs that specific existing in the disease network. Then calculate the top 6 genes with the highest degree in these gene pairs. For one sample, we have constructed the SSN, and calculated the top 10 genes with the highest degree in SSN. If the top 10 gens have less than 2 genes of the top 6 genes, the sample will be regarded as the normal sample. Otherwise, the sample can be regarded as the disease sample. The accuracy of the SSDN classification is 88.56%. On the other hand, we use the SSN method to classify the control sample and disease sample. We have selected the gene pairs that existing in the control network, and calculate the top 6 genes with the highest degree in these gene pairs. Then we have constructed the SSN in control and SSN in disease, and calculated the top 10 genes with the highest degree in SSN. If the top 10 genes have less than 2 genes of the top 6 genes, the sample will be regarded as the control sample, otherwise it’s a disease sample. Finally, we have selected the gene pair that existing in the disease network, and calculate the top 6 genes with the highest degree in these gene pairs. Based on the top 6 genes, we classify again. As a result, for BRCA, the accuracy of SSN in control classification is 54.40%, the accuracy of SSN in disease classification is 85.27%. The accuracy of classification for the other three cancer are also shown in the Table S4.

The result shows, the SSDN method select is indeed the specific gene in the disease network, and is better than SSN method. Because the SSN method can only reflect the information of a single individual, and our method can maximize the use of normal sample and disease sample.

**Note S7: Survival analysis compare with SSN method**

We compared the survival analysis with the SSN method. For BRCA and LIHC, the repetition hub genes were identified based on the top 10 hub genes of each DSSN, and the most frequent 10 repetition hub genes were used to survival analysis for tumor samples. We calculate the top 6 genes with the highest degree in SSN in control, and used the 10 repetition hub genes to divide tumor samples into two groups. One included the samples that there were at least 2 repetition hub genes to be in the top 6 hub genes of this sample; another included the samples that had less than 2 repetition hub genes to be in the top 10 hub genes of this sample. In the same way, we calculate the top 6 genes with the highest degree in SSN in disease, and repeat the same classification process above.

The p-value in DSSN method are below 0.05, is better than SSN (Table S5). It further illustrates that SSDN/DSSN method can find the difference between disease and control samples.

**TABLE AND FIGURES**

**Table S1**: For Disease network and Control network, we selected the most repetition 300, 100, 50, 30, 20 and 10 hub genes, compared whether these have common genes under these two reference networks by six ways.

| Database Network types | GSE27342 | GSE33335 | GSE63089 |
| --- | --- | --- | --- |
| specific genes in Disease and Control top-10 | 50.00% | 52.60% | 50.89% |
| specific genes in Disease and Control top-20 | 50.70% | 58.90% | 51.56% |
| specific genes in Disease and Control top-30 | 51.55% | 62.13% | 52.07% |
| specific genes in Disease and Control top-50 | 51.77% | 68.69% | 57.93% |
| specific genes in Disease and Control top-100 | 52.54% | 65.16% | 58.36% |
| specific genes in Disease and Control top-300 | 56.34% | 67.52% | 60.43% |
| common genes in Disease and Cntrol top-10 | 54.77% | 66.00% | 45.78% |
| common genes in Disease and Control top-20 | 57.67% | 64.00% | 47.22% |
| common genes in Disease and Control top-30 | 59.11% | 66.80% | 49.19% |
| common genes in Disease and Control top-50 | 56.51% | 69.39% | 52.89% |
| common genes in Disease and Control top-100 | 61.79% | 72.08% | 57.38% |
| common genes in Disease and Control top-300 | 62.76% | 75.19% | 63.76% |
| specific genes in Control top-10 | 54.93% | 57.60% | 56.00% |
| specific genes in Control top-20 | 55.99% | 62.80% | 57.40% |
| specific genes in Control top-30 | 58.83% | 67.00% | 59.92% |
| specific gene in Control top-50 | 59.61% | 66.00% | 61.10% |
| specific genes in Control top-100 | 66.30% | 70.00% | 68.93% |
| specific genes in Control top-300 | 70.87% | 74.13% | 70.85% |
| specific genes in Disease top-10 | 54.51% | 56.80% | 54.22% |
| specific genes in Disease top-20 | 56.62% | 61.60% | 57.78% |
| specific genes in Disease top-30 | 56.39% | 65.80% | 57.41% |
| specific genes in Disease top-50 | 61.92% | 64.96% | 58.04% |
| specific genes in Disease top-100 | 62.65% | 67.48% | 69.11% |
| specific genes in Disease top-300 | 64.80% | 69.13% | 68.94% |
| genes in Control top-10 | 53.94% | 68.40% | 53.56% |
| genes in Control top-20 | 57.18% | 66.00% | 57.22% |
| genes in Control top-30 | 57.42% | 67.87% | 58.89% |
| genes in Control top-50 | 58.82% | 68.40% | 59.78% |
| genes in Control top-100 | 68.96% | 70.64% | 62.33% |
| genes in Control top-300 | 70.31% | 75.32% | 68.19% |
| genes in Disease top-10 | 57.04% | 63.60% | 58.44% |
| genes in Disease top-20 | 59.08% | 62.40% | 60.33% |
| genes in Disease top-30 | 58.87% | 60.13% | 60.67% |
| genes in Disease top-50 | 65.11% | 62.96% | 60.76% |
| genes in Disease top-100 | 67.24% | 63.76% | 61.96% |
| genes in Disease top-300 | 70.02% | 68.72% | 65.75% |

**Table S2** The enrichment in CGC database compared with our method and previous method

| BRCA | top-10 | top-20 | top-30 | top-50 | top-100 |
| --- | --- | --- | --- | --- | --- |
| Our method | 99.82% | 100.00% | 100.00% | 100.00% | 100.00% |
| Previous method | 100.00% | 100.00% | 100.00% | 100.00% | 100.00% |
| LUAD | top-10 | top-20 | top-30 | top-50 | top-100 |
| Our method | 99.62% | 100.00% | 100.00% | 100.00% | 100.00% |
| Previous method | 96.76% | 88.72% | 98.96% | 99.99% | 100.00% |
| LUSC | top-10 | top-20 | top-30 | top-50 | top-100 |
| Our method | 100.00% | 100.00% | 100.00% | 100.00% | 100.00% |
| Previous method | 100.00% | 100.00% | 100.00% | 100.00% | 100.00% |
| LIHC | top-10 | top-20 | top-30 | top-50 | top-100 |
| Our method | 98.92% | 99.73% | 100.00% | 100.00% | 100.00% |
| Previous method | 74.54% | 88.69% | 99.98% | 100.00% | 100.00% |

**Table S3** The enrichment in KEGG database compared with our method and previous method

| BRCA | top-10 | top-20 | top-30 | top-50 | top-100 |
| --- | --- | --- | --- | --- | --- |
| Our method | 95.64% | 99.64% | 99.99% | 100% | 100% |
| Previous method | 100% | 100% | 100% | 100% | 100% |
| LUAD | top-10 | top-20 | top-30 | top-50 | top-100 |
| Our method | 96.81% | 95.68% | 99.81% | 100% | 100% |
| Previous method | 97.36% | 93.46% | 94.94% | 93.79% | 98.56% |
| LUSC | top-10 | top-20 | top-30 | top-50 | top-100 |
| Our method | 98.41% | 97.21% | 99.20% | 100% | 100% |
| Previous method | 98.66% | 97.42% | 96.18% | 100% | 100% |
| LIHC | top-10 | top-20 | top-30 | top-50 | top-100 |
| Our method | 100.00% | 100% | 100% | 100% | 100% |
| Previous method | 100.00% | 100% | 100% | 100% | 100% |

**Table S4** The accuracy of the classification for four cancer.

|  | SSDN | SSN in control | SSN in disease |
| --- | --- | --- | --- |
| BRCA | 88.56% | 54.40% | 85.27% |
| LUAD | 96.45% | 73.31% | 89.86% |
| LUSC | 98.37% | 88.02% | 96.91% |
| LIHC | 91.45% | 71.97% | 83.14% |

**Table S5** Survival analysis for SSN

| p-value | SSN in control | SSN in disease |
| --- | --- | --- |
| BRCA | 0.17 | 0.098 |
| LIHC | 0.01 | 0.5 |


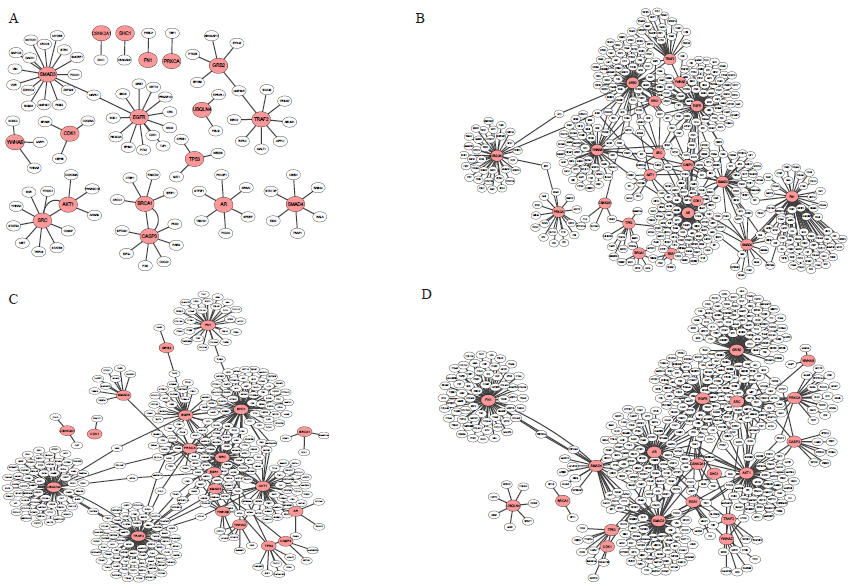


**Figure S1**. **The network modules with the potential disease modules in BRCA Control network and Disease network.** (A) The network modules among the top- 20 hub gene in Control network. (B) The network modules among the top- 20 hub gene in Disease network in sample BRCA_A0T6. (C) The network modules among the top- 20 hub gene in Disease network in sample BRCA_A4RY. (D) The network modules among the top- 20 hub gene in Disease network in sample BRCA_A1IX.


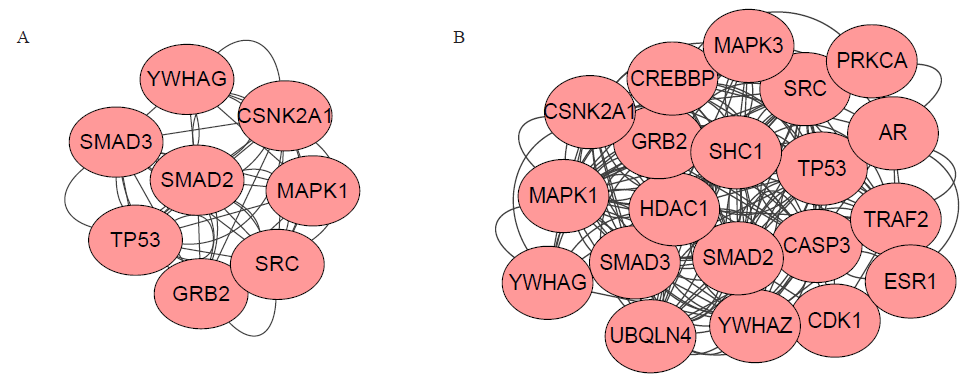


**Figure S2**. **The network modules with the potential disease modules in LIHC reference network.** (A) The network modules among the top- 10 hub genes. (B) The network modules among the top- 20 hub genes.


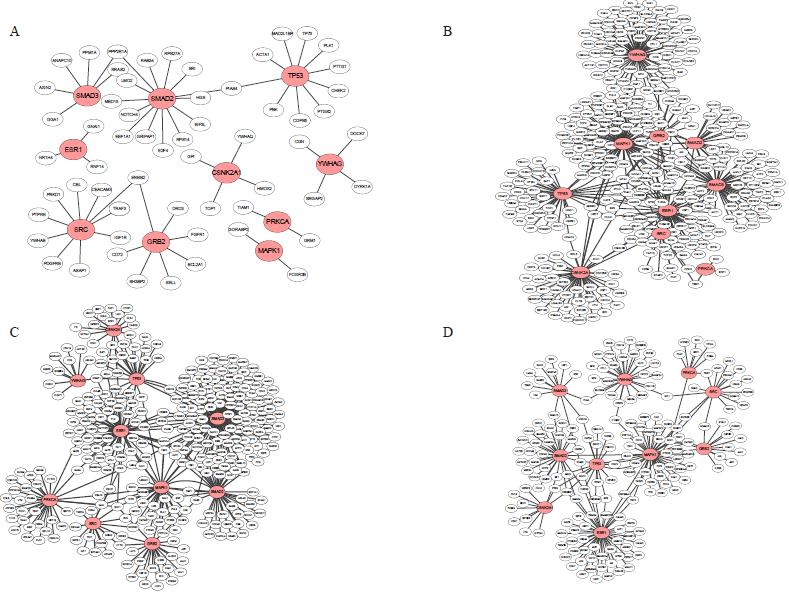


**Figure S3**. **The network modules with the potential disease modules in LIHC Control network and Disease network.** (A) The network modules among the top- 10 hub gene in Control network. (B) The network modules among the top- 10 hub gene in Disease network in sample LIHC_A9H1. (C) The network modules among the top- 10 hub gene in Disease network in sample LIHC _A69I. (D) The network modules among the top- 10 hub gene in Disease network in sample LIHC _AAC9.


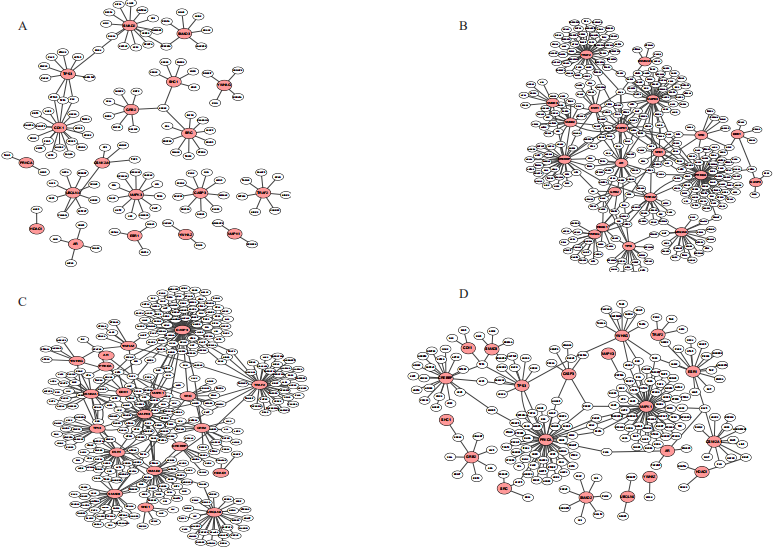


**Figure S4**. **The network modules with the potential disease modules in LIHC Control network and Disease network.** (A) The network modules among the top- 20 hub gene in Control network. (B) The network modules among the top- 20 hub gene in Disease network in sample LIHC_A110. (C) The network modules among the top- 20 hub gene in Disease network in sample LIHC _A520. (D) The network modules among the top- 20 hub gene in Disease network in sample LIHC _AA0V.


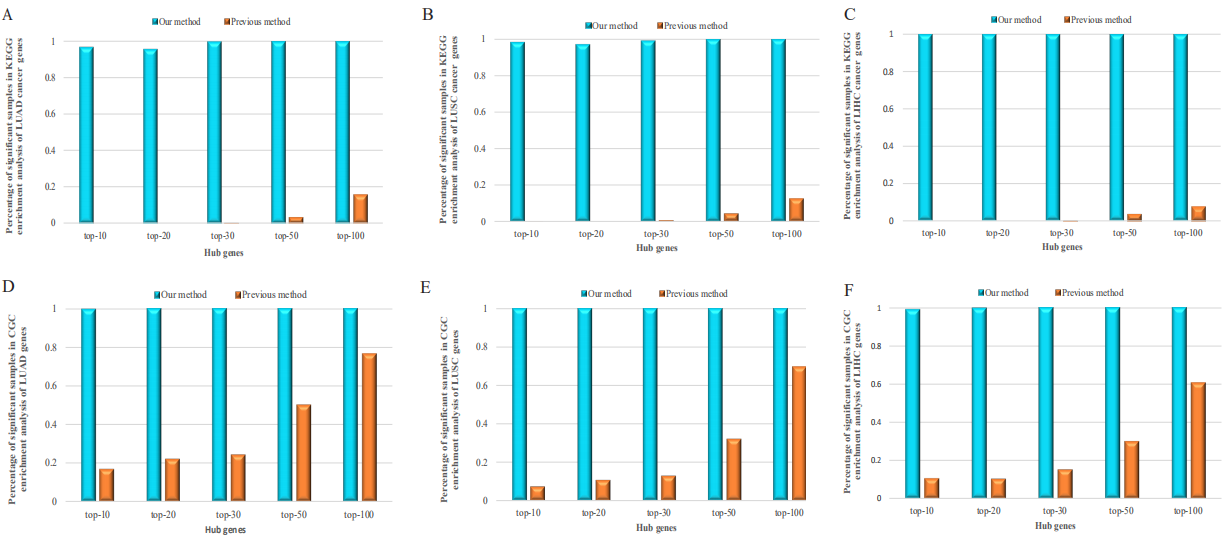


**Figure S5**. **The enrichment in KEGG pathway and CGC database compared with our method and SSN method.** (A)The proportion of significant samples in the enrichment analysis of top- 100, 50, 30, 20 and 10 highest degree genes for LUAD DSSN in the KEGG pathway and compare with the previous method. The x-axis is the hub genes and the y-axis is the percentage of significant samples in KEGG enrichment analysis. (B) The proportion of significant samples in the enrichment analysis of top- 100, 50, 30, 20 and 10 highest degree genes for LUSC DSSN in the KEGG pathway and compare with the previous method. The x-axis is the hub genes and the y-axis is the percentage of significant samples in KEGG enrichment analysis. (C) The proportion of significant samples in the enrichment analysis of top- 100, 50, 30, 20 and 10 highest degree genes for LIHC DSSN in the KEGG pathway and compare with the previous method. The x-axis is the hub genes and the y-axis is the percentage of significant samples in KEGG enrichment analysis. (D) The proportion of significant samples in the enrichment analysis of top- 100, 50, 30, 20 and 10 highest degree genes for LUAD DSSN in the CGC database and compare with the previous method. The x-axis is the hub genes of cancer and the y-axis is the percentage of significant samples in CGC enrichment analysis. (E) The proportion of significant samples in the enrichment analysis of top- 100, 50, 30, 20 and 10 highest degree genes for LUSC DSSN in the CGC database and compare with the previous method. The x-axis is the hub genes of cancer and the y-axis is the percentage of significant samples in CGC enrichment analysis. (F) The proportion of significant samples in the enrichment analysis of top- 100, 50, 30, 20 and 10 highest degree genes for LIHC DSSN in the CGC database and compare with the previous method. The x-axis is the hub genes of cancer and the y-axis is the percentage of significant samples in CGC enrichment analysis.


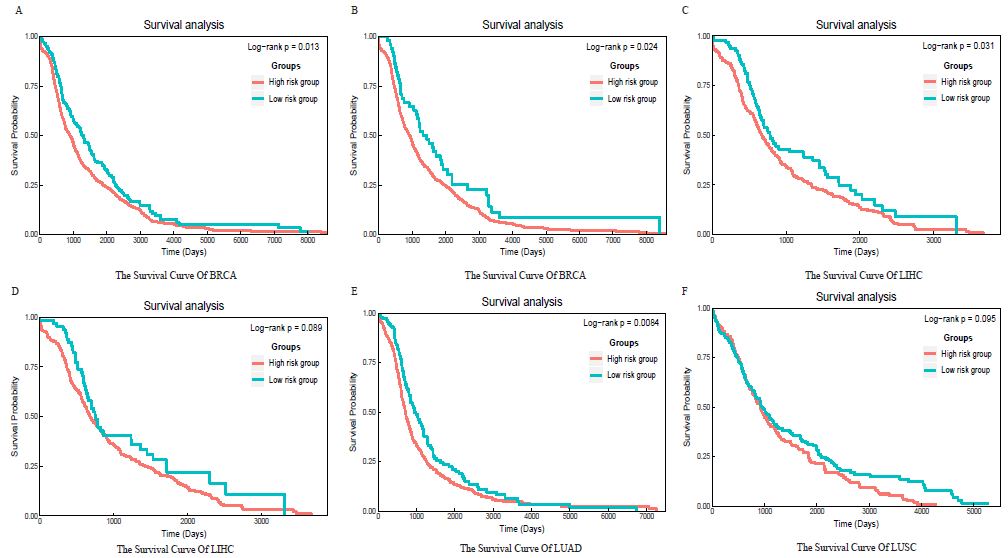


**Figure S6**. **Survival curve for BRCA and LIHC.** (A) Survival curve for BRCA survival analysis when using the most frequent 30 repetition hub genes to divide tumor samples into two groups. (B) Survival curve for BRCA survival analysis when using the most frequent 50 repetition hub genes to divide tumor samples into two groups. (C) Survival curve for LIHC survival analysis when using the most frequent 30 repetition hub genes to divide tumor samples into two groups. (D) Survival curve for LIHC survival analysis when using the most frequent 50 repetition hub genes to divide tumor samples into two groups. (E) Survival curve for LUAD survival analysis when using the most frequent 30 repetition hub genes to divide tumor samples into two groups. (F) Survival curve for LUSC survival analysis when using the most frequent 20 repetition hub genes to divide tumor samples into two groups.

**Reference**

1. Liu, X.; Wang, Y.; Ji, H.; Aihara, K.; Chen, L., Personalized characterization of diseases using sample-specific networks. *Nucleic Acids Research* **2016,** *44* (22), e164-e164.

2. Liu, X.; Chang, X., Identifying module biomarkers from gastric cancer by differential correlation network. *Oncotargets & Therapy* **2016,** *9*, 5701-5711.
