# Supplementary material for: Identifying network biomarkers of cancer by sample-specific differential network
Source: BMC Bioinformatics. 2022 Jun 15;23:230. doi: 10.1186/s12859-022-04772-1 (PMC9202129; doi:10.1186/s12859-022-04772-1)
Supplement: Supplementary file 16 — Additional file 16. Table S1. For Disease network and Control network, we selected the most repetition 300, 100, 50, 30, 20 and 10 hub genes, compared whether these have common genes under these two reference networks by six ways. [file 12859_2022_4772_MOESM16_ESM.docx]

**Supplement Table S1**

Three gastric cancer databases from the GEO database and these datasets are GSE33335, GSE63089, and GSE27342. We used normal samples from GSE63089 and GSE27342 to construct two reference networks, add every sample in the three datasets to the reference networks to construct sample-specific networks, respectively. For Disease network and Control network, there are six ways to obtain SSDN: constructing based on specific genes in Disease and Control networks, based on common genes in Disease and Control network, specific genes in Control networks, specific genes in Disease networks, genes only in Control networks, genes only in Disease networks. Then we selected the most frequent 300, 100, 50, 30, 20 and 10 repetition hub genes, and calculated the percentage of common hub genes based on two different reference networks.

| Database Network types | GSE27342 | GSE33335 | GSE63089 |
| --- | --- | --- | --- |
| specific genes in Disease and Control top-10 | 50.00% | 52.60% | 50.89% |
| specific genes in Disease and Control top-20 | 50.70% | 58.90% | 51.56% |
| specific genes in Disease and Control top-30 | 51.55% | 62.13% | 52.07% |
| specific genes in Disease and Control top-50 | 51.77% | 68.69% | 57.93% |
| specific genes in Disease and Control top-100 | 52.54% | 65.16% | 58.36% |
| specific genes in Disease and Control top-300 | 56.34% | 67.52% | 60.43% |
| common genes in Disease and Cntrol top-10 | 54.77% | 66.00% | 45.78% |
| common genes in Disease and Control top-20 | 57.67% | 64.00% | 47.22% |
| common genes in Disease and Control top-30 | 59.11% | 66.80% | 49.19% |
| common genes in Disease and Control top-50 | 56.51% | 69.39% | 52.89% |
| common genes in Disease and Control top-100 | 61.79% | 72.08% | 57.38% |
| common genes in Disease and Control top-300 | 62.76% | 75.19% | 63.76% |
| specific genes in Control top-10 | 54.93% | 57.60% | 56.00% |
| specific genes in Control top-20 | 55.99% | 62.80% | 57.40% |
| specific genes in Control top-30 | 58.83% | 67.00% | 59.92% |
| specific gene in Control top-50 | 59.61% | 66.00% | 61.10% |
| specific genes in Control top-100 | 66.30% | 70.00% | 68.93% |
| specific genes in Control top-300 | 70.87% | 74.13% | 70.85% |
| specific genes in Disease top-10 | 54.51% | 56.80% | 54.22% |
| specific genes in Disease top-20 | 56.62% | 61.60% | 57.78% |
| specific genes in Disease top-30 | 56.39% | 65.80% | 57.41% |
| specific genes in Disease top-50 | 61.92% | 64.96% | 58.04% |
| specific genes in Disease top-100 | 62.65% | 67.48% | 69.11% |
| specific genes in Disease top-300 | 64.80% | 69.13% | 68.94% |
| genes in Control top-10 | 53.94% | 68.40% | 53.56% |
| genes in Control top-20 | 57.18% | 66.00% | 57.22% |
| genes in Control top-30 | 57.42% | 67.87% | 58.89% |
| genes in Control top-50 | 58.82% | 68.40% | 59.78% |
| genes in Control top-100 | 68.96% | 70.64% | 62.33% |
| genes in Control top-300 | 70.31% | 75.32% | 68.19% |
| genes in Disease top-10 | 57.04% | 63.60% | 58.44% |
| genes in Disease top-20 | 59.08% | 62.40% | 60.33% |
| genes in Disease top-30 | 58.87% | 60.13% | 60.67% |
| genes in Disease top-50 | 65.11% | 62.96% | 60.76% |
| genes in Disease top-100 | 67.24% | 63.76% | 61.96% |
| genes in Disease top-300 | 70.02% | 68.72% | 65.75% |
