# Supplementary material for: Identifying network biomarkers of cancer by sample-specific differential network
Source: BMC Bioinformatics. 2022 Jun 15;23:230. doi: 10.1186/s12859-022-04772-1 (PMC9202129; doi:10.1186/s12859-022-04772-1)
Supplement: Supplementary file 17 — Additional file 17. Table S2. The enrichment in CGC database compared with our method and previous method. [file 12859_2022_4772_MOESM17_ESM.docx]

**Table S2** A comparison of the CGC enrichment analyses between our method and the SSN method

| BRCA | top-10 | top-20 | top-30 | top-50 | top-100 |
| --- | --- | --- | --- | --- | --- |
| Our method | 99.82% | 100.00% | 100.00% | 100.00% | 100.00% |
| Previous method | 100.00% | 100.00% | 100.00% | 100.00% | 100.00% |
| sDNB method | 94.57% | 96.42% | 100% | 100% | 100% |
| LUAD | top-10 | top-20 | top-30 | top-50 | top-100 |
| Our method | 99.62% | 100.00% | 100.00% | 100.00% | 100.00% |
| Previous method | 96.76% | 88.72% | 98.96% | 99.99% | 100.00% |
| sDNB method | 94.23% | 95% | 90.12% | 91.15% | 96.56% |
| LUSC | top-10 | top-20 | top-30 | top-50 | top-100 |
| Our method | 100.00% | 100.00% | 100.00% | 100.00% | 100.00% |
| Previous method | 100.00% | 100.00% | 100.00% | 100.00% | 100.00% |
| sDNB method | 97.67% | 100% | 100% | 100% | 100% |
| LIHC | top-10 | top-20 | top-30 | top-50 | top-100 |
| Our method | 98.92% | 99.73% | 100.00% | 100.00% | 100.00% |
| Previous method | 74.54% | 88.69% | 99.98% | 100.00% | 100.00% |
| sDNB method | 100.00% | 100% | 100% | 100% | 100% |
