# Supplementary material for: Identifying network biomarkers of cancer by sample-specific differential network
Source: BMC Bioinformatics. 2022 Jun 15;23:230. doi: 10.1186/s12859-022-04772-1 (PMC9202129; doi:10.1186/s12859-022-04772-1)
Supplement: Supplementary file 18 — Additional file 18. Table S3. The enrichment in KEGG database compared with our method and previous method [file 12859_2022_4772_MOESM18_ESM.docx]

**Table S3** A comparison of the KEGG enrichment analyses between our method and the SSN method.

| BRCA | top-10 | top-20 | top-30 | top-50 | top-100 |
| --- | --- | --- | --- | --- | --- |
| Our method | 95.64% | 99.64% | 99.99% | 100% | 100% |
| Previous method | 100% | 100% | 100% | 100% | 100% |
| sDNB method | 95.47% | 96% | 97.32% | 94.56% | 96.21% |
| LUAD | top-10 | top-20 | top-30 | top-50 | top-100 |
| Our method | 96.81% | 95.68% | 99.81% | 100% | 100% |
| Previous method | 97.36% | 93.46% | 94.94% | 93.79% | 98.56% |
| sDNB method | 96.89% | 97.54% | 96.23% | 97.56% | 97.6 |
| LUSC | top-10 | top-20 | top-30 | top-50 | top-100 |
| Our method | 98.41% | 97.21% | 99.20% | 100% | 100% |
| Previous method | 98.66% | 97.42% | 96.18% | 100% | 100% |
| sDNB method | 97.12% | 98.43% | 97.57% | 99.35% | 99.11% |
| LIHC | top-10 | top-20 | top-30 | top-50 | top-100 |
| Our method | 100.00% | 100% | 100% | 100% | 100% |
| Previous method | 100.00% | 100% | 100% | 100% | 100% |
| sDNB method | 100.00% | 100% | 100% | 100% | 100% |
