# Supplementary material for: Identifying network biomarkers of cancer by sample-specific differential network
Source: BMC Bioinformatics. 2022 Jun 15;23:230. doi: 10.1186/s12859-022-04772-1 (PMC9202129; doi:10.1186/s12859-022-04772-1)
Supplement: Supplementary file 19 — Additional file 19. Table S4. The accuracy of the classification for four cancer. [file 12859_2022_4772_MOESM19_ESM.docx]

**Table S4** The accuracy of the classification for four cancer.

|  | SSDN | SSN in control | SSN in disease |
| --- | --- | --- | --- |
| BRCA | 88.56% | 54.40% | 85.27% |
| LUAD | 96.45% | 73.31% | 89.86% |
| LUSC | 98.37% | 88.02% | 96.91% |
| LIHC | 91.45% | 71.97% | 83.14% |
