# Supplementary material for: Identifying network biomarkers of cancer by sample-specific differential network
Source: BMC Bioinformatics. 2022 Jun 15;23:230. doi: 10.1186/s12859-022-04772-1 (PMC9202129; doi:10.1186/s12859-022-04772-1)
Supplement: Supplementary file 20 — Additional file 20. Table S5. Survival analysis for SSN. [file 12859_2022_4772_MOESM20_ESM.docx]

**Table S5** Survival analysis for SSN

| p-value | SSN in control | SSN in disease |
| --- | --- | --- |
| BRCA | 0.17 | 0.098 |
| LIHC | 0.01 | 0.5 |
